# Supplementary material for: Spatial Configurations of 3D Extracellular Matrix Collagen Density and Anisotropy Simultaneously Guide Angiogenesis
Source: PLoS Comput Biol. 2023 Oct 23;19(10):e1011553. doi: 10.1371/journal.pcbi.1011553 (PMC10621972; doi:10.1371/journal.pcbi.1011553)
Supplement: S1 Text — The supporting information contains additional explanations and details of algorithmic updates to AngioFE. We have also included parameter and sensitivity studies that we used to guide finite element meshing and parameter selection. Finally, a table is provided with the coefficients used for various parameters in our simulations. (DOCX) [file pcbi.1011553.s001.docx]

Supporting Information: Spatial Configurations of 3D Extracellular Matrix Collagen Density and Anisotropy Simultaneously Guide Angiogenesis

Short title: Matrix Anisotropy and Density Simultaneously Guide Angiogenesis

Steven A. LaBelle^1,2^, A. Marsh Poulson IV^2^, Steve A. Maas^1,2^, Adam Rauff^1,2^, Gerard A. Ateshian^3^, Jeffrey A. Weiss^1,2*^

^1^ Department of Biomedical Engineering, University of Utah, Salt Lake City, UT

^2^ Scientific Computing and Imaging Institute, University of Utah, Salt Lake City, UT

^3^ Department of Mechanical Engineering, Columbia University, New York, NY

*Corresponding Author:

Jeffrey A. Weiss, PhD

Department of Biomedical Engineering

University of Utah

72 South Central Campus Drive

Salt Lake City, UT 84112

jeff.weiss@utah.edu

### Supporting Methods A: Linear interpolation between vectors

We simulated microvessel guidance based on directional vectors representing persistence cues and local matrix cues (fibril orientation). Previously we calculated the interpolation between two unit directional vectors as a weighted sum:

$\psi_{new}=\alpha\theta+(1-\alpha)\psi$,

where the parameter *α* ∈ [0, 1] controlled the balance between persistence (continuing along the same direction) and matrix cues. With this approach, the direction between ***θ*** and ***ψ*** does not vary linearly with *α*. We present a new method to enforce linear interpolation of the angle between to unit directional vectors:

.

The rotation ***R***(***θ***,***ψ***,*α*) occurred about the axis ***u*** = ***ψ*** × ***θ*,** which is mutually orthogonal to the persistence and matrix cue directions. This performed a coplanar rotation between the persistence and guidance directions. The coplanar rotation tensor was defined by the Rodrigues rotation formula (1, 2). The parameter *α* scaled *ϕ*, the angle between persistence and guidance.

,

where ⊗ is the outer product. This new approach is visualized in S1 Fig. To ensure tips did not grow backwards, a check was performed via the dot product of the accepted direction and the persistence direction:

$\text{if }\theta\cdot\psi<0,\text{ then }\theta=-\theta$.

### Supporting Methods B: Sensitivity study – collage orientation weight (*α*)

A study was performed to determine how vector field and EFD field approaches were sensitive to the collagen orientation weight parameter *α*. This parameter was varied between 0.0 and 1.0 which covers extreme cases where vessel growth occurs along a straight line (persistence, *α* = 0.0) or vessel growth occurs entirely along the collagen direction (haptotaxis, *α* = 1.0). Sensitivity was assessed by calculating the ratio of the first and second semiprincipal axes of the microvessel ODF after 10 days of growth (S2 Fig). The discrete fiber approach was unable to predict the degree of alignment seen experimentally for medium and high anisotropy regardless of density. We believe this may be related to our finding in the previous section that vector field approaches appear to be constrained by the underlying collagen ODF. In contrast, the EFD was able to match the range of experimental data for all cases studied.

### Supporting Methods C: Collagen fibril strength dependence

Our results from the parametric study on the collagen fibril strength (*α*) indicated that the collagen strength increased quickly with matrix anisotropy. A sigmoidal function was developed to link *α* to the 2D fractional anisotropy (FA) based results from our numerical experiments:

$\alpha(\text{FA})=\alpha_{0}+\frac{a_{\alpha}(\rho)}{1+e^{b_{\alpha}\left(\text{FA}-c_{\alpha}\right)}}$,

$a_{\alpha}(\rho)=0.05\left(\rho-2.0\right);\text{ 0.05 }\leq a_{\alpha}\leq0.30$.

Here, only the 2D fractional anisotropy was calculated between the primary and secondary semiprincipal axes of the EFD. This was done because the 3D anisotropy can be greatly affected by relatively small minor semiprincipal axes. For instance, the anisotropy of a low-density low-aligned gel ranged between 0.53-0.65, while the 2D anisotropy was 0.23. Further, similar effects of alignment have been seen in 2D and 3D *in vitro* (3, 4). The magnitude of the amplitude of the sigmoidal curve, *a_α_*(*ρ*), varied slightly with matrix density based on calibration simulation results (Supporting Methods B, S2 Fig). The amplitude was capped at a value of 0.3 since values of *α*(FA) above 0.4 yielded highly tortuous vessels (S14 Fig). This relationship was implemented in AngioFE for all predictive simulations.

### Supporting Methods D: Vector field and EFD approaches were insensitive to mesh refinement

A mesh refinement study was performed to determine the sensitivity of vector field and EFD approaches to the spatial discretization of the finite element mesh. Sensitivity was assessed by calculating the ratio of the first and second semiprincipal axes of the microvessel XY ODF after 10 days of growth. The ODF axis ratio gradually increased with mesh refinement for both the discrete fiber and continuous EFD approach (S4 Fig). The vector field approach was unable to predict the degree of alignment seen experimentally even with mesh refinement. We believe this may be related to our finding in the previous section that vector field approaches appear to be constrained by the underlying collagen ODF. In contrast, the EFD approach was accurate for all meshes studied. This result is not surprising since the same EFD will be represented regardless of mesh refinement. The ODF axis ratio does begin to exceed experimental values for highly-refined meshes with side lengths less than 50 µm. However this occurs because shorter timesteps are taken with refined meshes since vessels are encountering element-element boundaries more frequently (Supporting Methods 8). In the future, vessel sampling frequency will have a minimum time-step required before reorientation is allowed to occur.

### Supporting Methods E: Pseudo-deformation validation deformations

Pseudo-deformed EFDs were compared to “true” ODF deformation. Uniaxial tension-compression, biaxial tension-compression, simple shear, and pure shear were studied to determine how different modes of deformation affected the accuracy of the pseudo-deformation (S5-S8 Figs). Below are the deformation gradients ***F*** for each mode that was studied. All deformations were assumed to be isochoric. The MATLAB script used for these analyses is available in the AngioFE GitHub repository documentation ([github.com/febiosoftware/AngioFE](https://github.com/febiosoftware/AngioFE)).

Uniaxial Tension or Compression:

$$\left[F\right]=\left[\begin{matrix} \lambda& 0 & 0 \\ 0 & \frac{1}{\sqrt{\lambda}} & 0 \\ 0 & 0 & \frac{1}{\sqrt{\lambda}} \end{matrix}\right]$$

Biaxial Tension or Compression:

$$\left[F\right]=\left[\begin{matrix} \lambda_{1} & 0 & 0 \\ 0 & \lambda_{2} & 0 \\ 0 & 0 & \frac{1}{\lambda_{1}\lambda_{2}} \end{matrix}\right]$$

*For this case, we considered strip biaxial tension and compression where *λ*_2_ = 1.

Simple Shear:

$$\left[F\right]=\left[\begin{matrix} 1 & \kappa& 0 \\ 0 & 1 & 0 \\ 0 & 0 & 1 \end{matrix}\right]$$

Pure Shear:

$$\left[F\right]=\left[\begin{matrix} 1 & \frac{\kappa}{2} & 0 \\ \frac{\kappa}{2} & 1 & 0 \\ 0 & 0 & \frac{1}{1-\frac{\kappa^{2}}{4}} \end{matrix}\right]$$

### Supporting Methods F: Vector field simulations of growth in anisotropy gradients and tumor associated collagen signatures (TACS)

The anisotropy gradient and TACS simulations were performed using the vector field representation of collagen orientation to assess differences between predictions by vector field and EFD field simulations. These simulations did not account for the degree of anisotropy since it is not reliably derived from the 8 finite element nodes. Instead, the legacy growth rule from prior versions of AngioFE was used (5, 6):

$\nu(\rho)=\nu_{0}+a_{0}\exp\left(-a_{1}\rho\right)$.

This equation was fit to growth data for microvessel growth in isotropic matrices of varying density.

### Supporting Methods G: Sigmoidal vessel growth rule

The network length over time in our *in vitro* models followed a sigmoidal curve (7). The growth length over time for a single fragment was thus determined from the time derivative of the fit to the sigmoidal curve:

$g(t)=g_{0}+\frac{a_{g}}{1+e^{-\frac{t-t_{1/2}}{b_{g}}}}$.

Notably, the amplitude of the sigmoidal curve, *a_g_*, was divided by the number of initial vessel fragments and then again by 2 since each vessel begins with 2 tips. The derivative of this is the growth rate :

$\frac{dg(t)}{dt}=\frac{a_{g}e^{-\frac{t-t_{1/2}}{b_{g}}}}{b_{g}\left(1+e^{-\frac{t-t_{1/2}}{b_{g}}}\right)^{2}}$.

### Supporting Methods H: Updated vessel initialization step for AngioFE3

AngioFE version 3.0 introduces a refined initialization step that accurately reproduced the initial morphology of seeded microvessel fragments. Previously, the initial vessel parent fragments were seeded as straight lines with random orientation and uniform length. However, in our experiments, these fragments had some initial alignment in the XY plane and were curved rather than straight (6). Further, the initial vessel length ranged from tens to hundreds of microns. The refined initialization step reproduced the orientation, length, and curvature observed on the first day of experimental cultures. During initialization, tips from the same parent fragment were seeded ~ 1 μm apart. These tips were simulated to extend until the distance between them reached the initial vessel length. The distance and direction each tip grew during initialization were sampled from experimentally-derived distributions of the initial microvessel length and orientation (S12 Fig). These distributions could be modified via user input to generate the conditions of the initial vasculature during various in vitro and in vivo experiments. For this study, initial microvessel lengths were based on measurements from gels 1 day after creation. The histogram of initial vessel lengths was fit to the rational function:

$P(L_{0})=\frac{p_{0}x+p_{1}}{x^{2}+q_{0}x+q_{1}}$.

The initial lengths were fixed in the range of 30 – 800 μm. The initial number of vessel fragments was set to match the initial experimental microvascular density (total length of microvessels per volume).

### Supporting Methods I: Ray-Tracing Vessel Growth Algorithm

Vessel tips were managed by the finite element containing their positions. The length a vessel could grow during the time step, *u_g_*, and direction, ***ψ*** were calculated. Then the following algorithm was used to determine vessel growth:

1. In the natural coordinate system, calculate the distance *u_f_* between the vessel tip and the element face the vessel projects onto.

2. If: *u_g_* < *u_f_*

The vessel will only grow in this element. Grow the tip to the new position.

Else:

If there is an element on the other face

Grow the vessel tip to the face and then pass it to the adjacent element.

Subtract the distance grown from *u_g_* and return to 1.

Else

Grow the vessel tip to the element face and subtract the distance grown from *u_g_*.

If the user specifies the bouncy condition

Bounce the vessel off the wall. This is done by calculating $\psi_{b}=\psi-2n(\psi\cdot n)$ where ***n*** is the normal vector of the boundary face and $n(\psi\cdot n)$ is the projection of ***ψ*** onto ***n***.

Else the vessel direction is along the face of the element

$\psi_{t}=\psi-n(\psi\cdot n)$, yielding the projection of ***ψ*** onto the element face.

Return to step 1.

### Supporting Methods J: Branching

Branching was specified using a distribution of the expected length to branch, i.e., how far a vessel would grow before branching. The direction that the new vessel departed from the parent vessel was governed by two angles – the zenith, and the azimuth (S13 Fig). The zenith was the angle directly between the parent vessel and the branch. The azimuth was a rotation of the zenith around the axis of the parent vessel. Each angle was sampled from a uniform distribution with the zenith angle ranging from 30º – 70º and the azimuth angle ranging from 0º – 360º.

### Supporting Methods K: Vessel volume fraction growth rule

Our prior growth rules were based on experimental cultures of microvessels in collagen gels. Microvascular growth in these experiments was characterized by a sigmoidal curve where microvascular growth increased during the first 6 days of culture and then decreased as gels became heavily vascularized. We previously modeled growth either by matching the total vascular length to a sigmoidal curve or the microvessel velocity to the derivative of a sigmoidal curve. However, these curves were based on *in vitro* measurements of growth which follow a different time-course than *in vivo* healing. Sigmoidal curves are used to model in vitro growth since growth attenuates as cells reach confluency and resources become competitive (i.e., space, nutrients, etc.) However, the healing process *in vivo* can occur over the span of weeks to months. Further, *in vivo* healing differs from *in vitro* growth since injured tissues may occupy much more space than in vitro constructs (and thus confluency may occur later in some regions than others). To simulate growth over longer periods of culture we developed an alternative growth velocity model. For this approach, the growth velocity (i.e., the length a vessel grows over a day) was prescribed by the sigmoidal curve

$g_{a}=s_{a}\frac{a_{a}}{1+e^{-\frac{t-c_{a}}{b_{a}}}}$,

where *a_a_* was the maximum distance a vessel could grow in a day, *t* was time, *c_a_* was the center of the curve, and *b* was the spread of the curve. This sigmoid curve was then scaled by *s_a_*, which was an exponential function based on the ratio of the current vascular volume fraction in the current finite element

$s_{a}=a_{s}e^{-\frac{q_{s}}{b_{s}}}+s_{0}$.

The variable *q_s_* was the ratio of the current vessel volume fraction *w_s_* to a threshold volume fraction *w_thresh_* which had a minimum at 1.0:

$q_{s}=\min\left(\frac{w_{v}}{w_{thresh}},1.0\right)$.

The values *a_s_*, *b_s_*, and *s_0_* were selected so that when the vessel volume fraction was below the threshold *s_a_* = 1.0. When *q_s_* > 1.0, *s_a_* rapidly decreased until *s_a_* = *s*_0_. Thus, vessel growth velocity *g_a_* rapidly reduced when the finite elemental vessel volume fraction exceeded the threshold *w_thresh_*, which was chosen based on vascularity in previous *in vitro* experiments and *in vivo* studies of ligament healing (8, 9). Vessel volumes were calculated assuming a cylindrical volume with constant radius of 6.3 µm. When the finite element vessel volume fraction was below the threshold, the growth rate reached a constant speed of *a_a_*. For the current studies, the value of *a_a_* was based on the maximum growth velocity from prior experiments of quick growing vessels (6, 7).

### Supporting Methods L: Sprout traction stresses

Traction fields were imposed around growing vessel tips which allowed them to deform their surrounding ECM (5, 10). The traction fields were projected to the finite element integration points as given by (5):

$\sigma_{s}(x,\rho)=a(t)\nu(\rho)e^{-b||r||}\cos^{N}\left(\frac{\psi_{x}}{2}\right)\hat{r}\otimes\hat{r}$.

Here, ***x*** is the position of the finite element integration point, ***r*** is the vector from the vessel tip to ***x***, and *ψ****_x_*** is the angle between ***ψ****_new_* and ***r***. The magnitude of traction increases with vessel growth as dictated by the time-sensitive sigmoid function *a*(*t*)

$a(t)=a_{0}+\frac{a_{amp}}{1+e^{-\frac{t-a_{center}}{a_{width}}}}$.

Tractions were also influenced by the density-dependent function ν(*ρ*):

$\nu(\rho)=\nu_{0}+\nu_{a}\exp(-\nu_{b}\rho)$.

Note that the traction magnitude is independent of anisotropy; this simplification was made due to a lack of experimental data. The parameter *b* influenced the falloff of stress with distance from the vessel tip. Finally, *N* influenced the degree of polarization in front of the vessel.

### Supporting Methods M: Sprout traction and fibril remodeling sensitivity study

Growing neovessels deform the matrix around them, causing reorientation of these fibrils. New growing vessels are simultaneously guided along the direction of the collagen fibril. Thus, we performed a sensitivity study to determine how the magnitude of the sprout traction affects growth in compliant anisotropic matrices. The same parameter study from Supporting Methods 2 (varying *α* and comparing Discrete/EFD approaches to experimental data) was reproduced 3 times with varying values of sprout traction *a_amp_* from Eq . We investigated a stress-free case (*a_amp_* = 0.0 μPa), the baseline case (*a_amp_* = 3.72 μPa, same value used throughout the manuscript and our prior publications), and a high-stress case (*a_amp_* = 37.2 μPa, 1 order magnitude greater than our baseline). Sensitivity was determined by evaluating differences in the ODF axis ratio of the resulting microvascular network.

Qualitative evaluation of the ODF axis ratios for each case indicates that in general, vascular networks are insensitive to stress at and below the baseline case. For high-stress cases, we see increased polarization of the microvascular network as indicated by higher values for the ODF axis ratio. The discrete simulations appear to differ more generally than the EFD cases. Additionally, EFD simulations in highly aligned matrices did not differ due to traction stress, indicating that more aligned matrices are less sensitive to vascular remodeling.

### S1 Text References

1. Rodrigues O. Des lois géométriques qui régissent les déplacements d'un système solide dans l'espace, et de la variation des coordonnées provenant de ces déplacements considérés indépendamment des causes qui peuvent les produire. Journal de Mathématiques Pures et Appliquées. 1840;1(5):380-440.

2. Taylor CJ, Kriegman DJ. Minimization on the Lie Group SO(3) and Related Manifolds. New Haven, CT: Center for Systems Science, Department of Electrical Engineering, Yale University; 1994. Contract No.: 9405.

3. McCoy MG, Wei JM, Choi S, Goerger JP, Zipfel W, Fischbach C. Collagen Fiber Orientation Regulates 3D Vascular Network Formation and Alignment. ACS Biomaterials Science & Engineering. 2018;4(8):2967-76.

4. LaBelle SA, Dinkins SS, Hoying JB, Budko EV, Rauff A, Strobel HA, et al. Matrix anisotropy promotes angiogenesis in a density-dependent manner. American Journal of Physiology-Heart and Circulatory Physiology. 2022.

5. Edgar LT, Hoying JB, Weiss JA. In Silico Investigation of Angiogenesis with Growth and Stress Generation Coupled to Local Extracellular Matrix Density. Ann Biomed Eng. 2015;43(7):1531-42.

6. Strobel HA, LaBelle SA, Krishnan L, Dale J, Rauff A, Poulson AM, et al. Stromal Cells Promote Neovascular Invasion Across Tissue Interfaces. Frontiers in Physiology. 2020;11(1026).

7. Edgar LT, Sibole SC, Underwood CJ, Guilkey JE, Weiss JA. A computational model of in vitro angiogenesis based on extracellular matrix fibre orientation. Comput Methods Biomech Biomed Engin. 2013;16(7):790-801.

8. Bray RC, Rangayyan RM, Frank CB. Normal and healing ligament vascularity: a quantitative histological assessment in the adult rabbit medial collateral ligament. J Anat. 1996;188 ( Pt 1)(Pt 1):87-95.

9. Underwood CJ, Edgar LT, Hoying JB, Weiss JA. Cell-generated traction forces and the resulting matrix deformation modulate microvascular alignment and growth during angiogenesis. Am J Physiol Heart Circ Physiol. 2014;307(2):H152-64.

10. Edgar LT, Maas SA, Guilkey JE, Weiss JA. A coupled model of neovessel growth and matrix mechanics describes and predicts angiogenesis in vitro. Biomech Model Mechanobiol. 2015;14(4):767-82.
