# Supplementary material for: Spatial Configurations of 3D Extracellular Matrix Collagen Density and Anisotropy Simultaneously Guide Angiogenesis
Source: PLoS Comput Biol. 2023 Oct 23;19(10):e1011553. doi: 10.1371/journal.pcbi.1011553 (PMC10621972; doi:10.1371/journal.pcbi.1011553)
Supplement: S1 Table — The coefficients used for various parameters in our simulations are presented in this table. Justifications and any additional assumptions are presented as well. (DOCX) [file pcbi.1011553.s002.docx]

### S1 Table : Simulation Parameters

| Parameter Name | Symbol | Value | Reference/Rationale |
| --- | --- | --- | --- |
| Parametric study | | | |
| Low anisotropy proto-EFD | (*β*_1_*^p^, β*_2_*^p^, β*_3_*^p^*) | (1.0,1.0,0.55) | (1) |
| Medium anisotropy proto-EFD | (*β*_1_*^p^, β*_2_*^p^, β*_3_*^p^*) | (1.0,1.0,0.35) | (1) |
| High anisotropy proto-EFD | (*β*_1_*^p^, β*_2_*^p^, β*_3_*^p^*) | (1.0,1.0,0.35) | (1) |
| Low-anisotropy low-density EFD | (*β*_1_*^0^, β*_2_*^0^, β*_3_*^0^*) | (1.2991,1.0,0.1) | (1) |
| Medium-anisotropy-low density EFD | (*β*_1_*^0^, β*_2_*^0^, β*_3_*^0^*) | (1.7732,1.0,0.5) | (1) |
| High-anisotropy low-density EFD | (*β*_1_*^0^, β*_2_*^0^, β*_3_*^0^*) | (2.7978,1.0,0.9) | (1) |
| Low-anisotropy high-density EFD | (*β*_1_*^0^, β*_2_*^0^, β*_3_*^0^*) | (1.2991,1.0,0.4) | (1) |
| Medium-anisotropy high-density EFD | (*β*_1_*^0^, β*_2_*^0^, β*_3_*^0^*) | (1.7732,1.0,0.9) | (1) |
| High-anisotropy high-density EFD | (*β*_1_*^0^, β*_2_*^0^, β*_3_*^0^*) | (2.7978,1.0,1.0) | (1) |
| Proto- collagen fibril weight | *α_p_* | 0.3 | (1) |
| Number of initial parent microvessel fragments | *n_frag_* | 130 | (1) |
| Range of initial parent microvessel lengths | *L_0_* | 30-800 µm | (1) |
| Collagen biphasic material properties | | | |
| Collagen elastic modulus | *E* | 31.9 Pa | (2, 3) |
| Collagen Poisson’s ratio | *ν* | 0.0 | (3) |
| Collagen fibril exponential power scalar | *α* | 0.0 | Set to 0 to match FEBio EFD material used previously (3). |
| Collagen fibril modulus | *ξ* | 4*πβ* × 550 Pa | (2). 4*πβ* factor converts EFD material parameters to a continuous fibril distribution with an exponential power law in FEBio. |
| Collagen fibril exponential | *β* | 2.5 | (3) |
| Collagen permeability | *κ* | 3.10 mm^3^ / kg ⋅ s | (2) |
| Initial solid volume fraction | Φ_0_ | 0.00567 | (2) |
| Vessel growth parameters | | | |
| *α*(FA) minimum | *α*_0_ | 0.1 | Calibration studies |
| *α*(FA) spread | *b_α_* | -33.28 | Calibration studies |
| *α*(FA) center | *c_α_* | 0.3 | Calibration studies |
| Lorentzian magnitude | *a_ν_* | 3.413 | Calibration studies |
| Lorentzian density center | *ρ*_0_ | 2.00 | Calibration studies |
| Lorentzian FA center | FA_0_ | 0.85 | Calibration studies |
| Lorentzian density spread | *b_ν_* | 1.759 | Calibration studies |
| Lorentzian FA spread | *c_ν_* | 0.6155 | Calibration studies |
| Lorentzian minimum | *d_ν_* | 0.10 | Calibration studies |
| Sigmoid growth max length | *a_g_* | 105 | (3) |
| Sigmoid growth spread  S | *b_g_* | 1.3 | (3) |
| Sigmoid growth center | *t*_1/2_ | 6 | (3) |
| Initial vessel length pdf | *p*_0_ | 0.106 | (1), S12 Fig |
| Initial vessel length pdf | *p*_1_ | 0.6905 | (1), S12 Fig |
| Initial vessel length pdf | *q*_0_ | -89.14 | (1), S12 Fig |
| Initial vessel length pdf | *q*_1_ | 2369 | (1), S12 Fig |
| Traction stress parameters | | | |
| Stress sigmoid min magnitude | *a*_0_ | 0 Pa | Assume cells cannot push outward. |
| Stress sigmoid magnitude | *a_amp_* | 3.72 μPa | (3, 4) |
| Stress sigmoid width | *a_width_* | 0.5435 days | (3, 4) |
| Stress sigmoid shift | *a_center_* | 7.0 days | (3, 4) |
| Stress density scaling | *ν*_0_ | -0.016 | (3, 4) |
| Stress density scaling | *ν_a_* | 5.1605 | (3, 4) |
| Stress density scaling | *ν_b_* | 0.5112 | (3, 4) |
| Stress range | *b* | 200.0 µm | (3, 4) |
| Fan exponential | *N* | 2.0 | (3, 4) |
| Predictive simulation: Anisotropy gradient | | | |
| Baseline EFDs | (*β*_1_, *β*_2_, *β*_3_) | (1.3,1.0,0.5) | Assumed |
| Vessel volume fraction growth max velocity | *a_a_* | 100.0 | Assumed |
| Vessel volume fraction growth spread | *b_a_* | 2.572 | Assumed |
| Vessel volume fraction growth center | *c_a_* | 5.0 | Assumed |
| Vessel volume fraction exponential scale | *a_s_* | 11.92 | Assumed |
| Vessel volume fraction exponential spread | *b_s_* | 0.4 | Assumed |
| Vessel volume fraction threshold | *w_thresh_* | 0.02 | (5) |
| Vessel volume fraction exponential scale minimum | *s*_0_ | 0.0215 | Assumed |
| Stress sigmoid center | *a_width_* | 9 days | Time increased for longer simulation |
| Stress sigmoid width | *b* | 0.143 days | Width increased for longer simulation |
| Predictive simulation: TACS structural interfaces | | | |
| Baseline, TACS-1 EFDs | (*β*_1_, *β*_2_, *β*_3_) | (1.3,1.0,0.3) | (4, 6) |
| TACS-2, TACS-1+2 EFDs | (*β*_1_, *β*_2_, *β*_3_) | (1.0,3.0,0.3) | (4, 6) |
| TACS-3, TACS-1+3 EFDs | (*β*_1_, *β*_2_, *β*_3_) | (3.0,1.0,0.3) | (4, 6) |
| Tumor and periphery density | *ρ_0_* | 3.0 mg/mL | (4, 6) |
| Baseline, TACS-2, TACS-3 interface density | *ρ_0_* | 3.0 mg/mL | (4, 6) |
| TACS-1, TACS-1+2, TACS-1+3 interface density | *ρ_0_* | 5.0 mg/mL | (4, 6) |

### S1 Table References

1. LaBelle SA, Dinkins SS, Hoying JB, Budko EV, Rauff A, Strobel HA, et al. Matrix anisotropy promotes angiogenesis in a density-dependent manner. American Journal of Physiology-Heart and Circulatory Physiology. 2022.

2. Ruehle MA, Eastburn EA, LaBelle SA, Krishnan L, Weiss JA, Boerckel JD, et al. Extracellular matrix compression temporally regulates microvascular angiogenesis. Sci Adv. 2020;6(34).

3. Edgar LT, Hoying JB, Weiss JA. In Silico Investigation of Angiogenesis with Growth and Stress Generation Coupled to Local Extracellular Matrix Density. Ann Biomed Eng. 2015;43(7):1531-42.

4. Strobel HA, LaBelle SA, Krishnan L, Dale J, Rauff A, Poulson AM, et al. Stromal Cells Promote Neovascular Invasion Across Tissue Interfaces. Frontiers in Physiology. 2020;11(1026).

5. Bray RC, Rangayyan RM, Frank CB. Normal and healing ligament vascularity: a quantitative histological assessment in the adult rabbit medial collateral ligament. J Anat. 1996;188 ( Pt 1)(Pt 1):87-95.

6. Brett EA, Sauter MA, Machens H-G, Duscher D. Tumor-associated collagen signatures: pushing tumor boundaries. Cancer & Metabolism. 2020;8(1):14.
