# Supplementary figures and images for: Spatial Configurations of 3D Extracellular Matrix Collagen Density and Anisotropy Simultaneously Guide Angiogenesis
Source: PLoS Comput Biol. 2023 Oct 23;19(10):e1011553. doi: 10.1371/journal.pcbi.1011553 (PMC10621972; doi:10.1371/journal.pcbi.1011553)

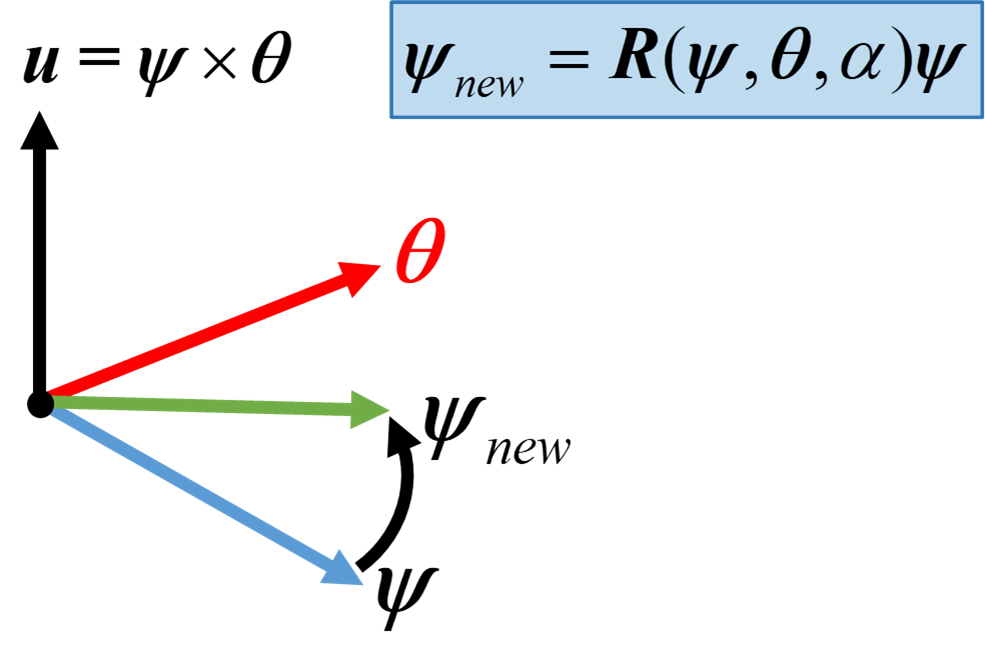

Supplement: S1 Fig — A vessel tip is centered at the origin. The new direction that a vessel grows depends on the previous direction (ψ) and the local collagen direction (θ). A coplanar rotation from ψ to θ occurs about the axis u = ψ × θ. The new direction ψnew is found by partially rotating ψ towards θ. The fraction of the whole rotation is determined by α ∈ [0, 1] where α = 0 performs no rotation, and α = 1 performs the entire rotation. (TIF) [file pcbi.1011553.s003.tif]

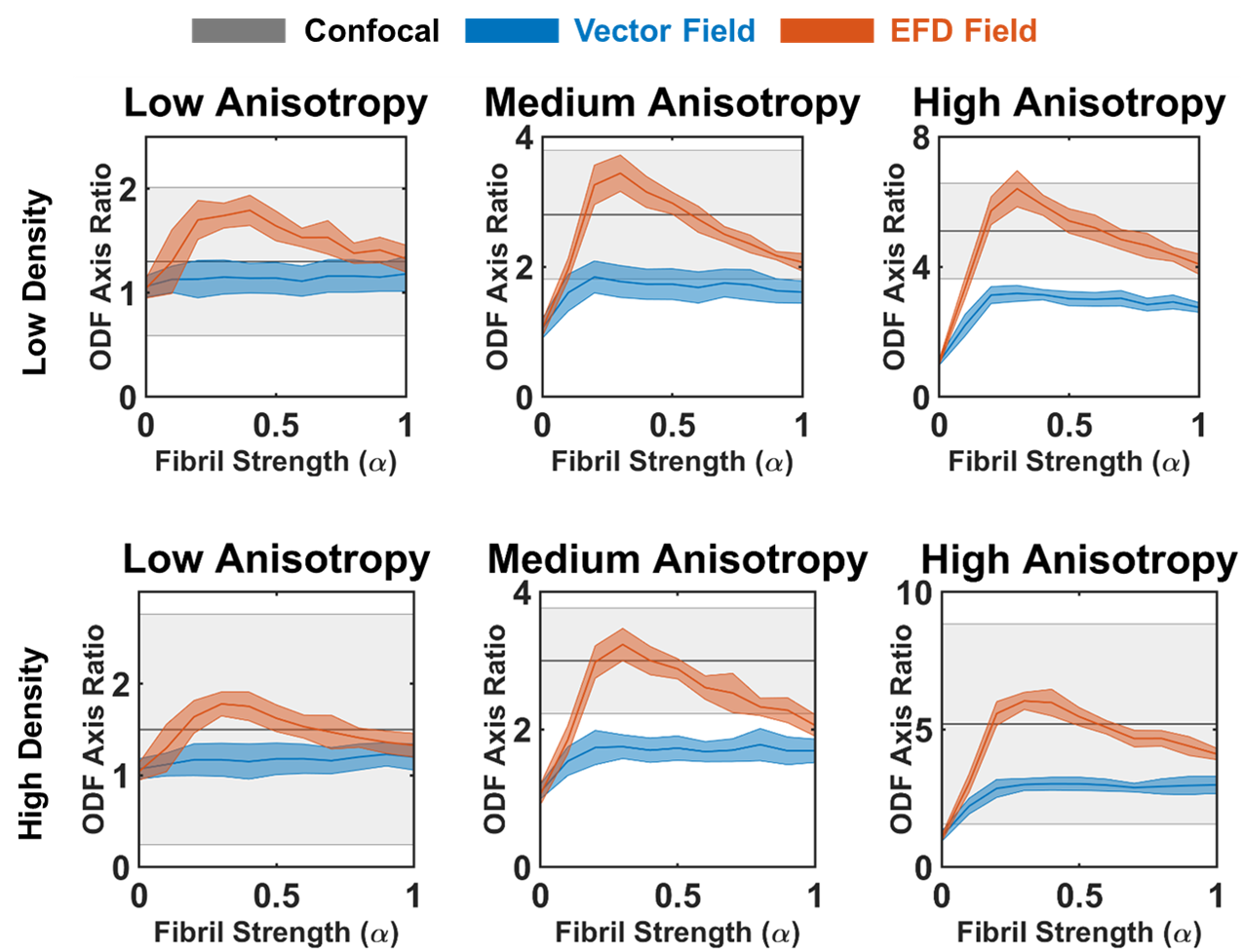

Supplement: S2 Fig — Sensitivity study–Collagen fibril orientation weight (α). Top: Low-density collagen results for simulations where the fibril weight (α) was varied from 0.0–1.0. Here, 0 indicates vessel grow along the persistence direction while 1 indicates that vessels grow entirely along the sampled fibril direction. Plotted is the ratio of the major and minor semiprincipal axes for ODFs of microvascular orientation. Discrete fibril approaches and continuous EFD approaches to representing matrix fibril distributions were compared to prior experimental measures (target) for simulations of growth in low, medium, or high anisotropy collagen. Bottom: Similar results were generated from simulations with high-density matrix collagen. (TIF) [file pcbi.1011553.s004.tif]

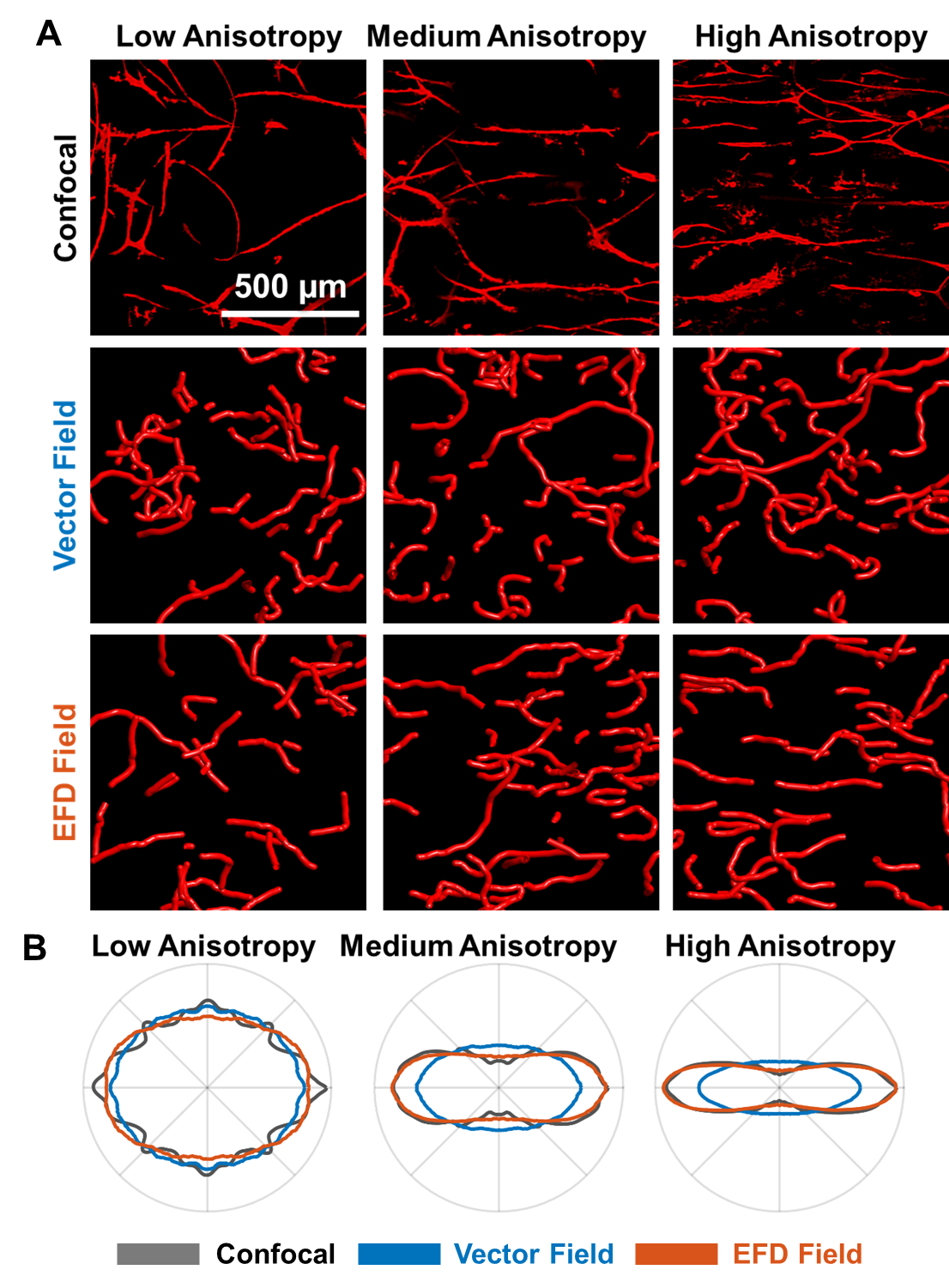

Supplement: S3 Fig — A. Z projections of experimental (confocal) and simulated microvascular networks grown in low, medium, or high anisotropy collagen. Depth of field = 200 μm. B. XY-plane ODFs for each experimental or simulated case. Overlap is seen between the confocal and EFD field ODFs for all cases. The presented results are for 4 mg/mL collagen. (TIF) [file pcbi.1011553.s005.tif]

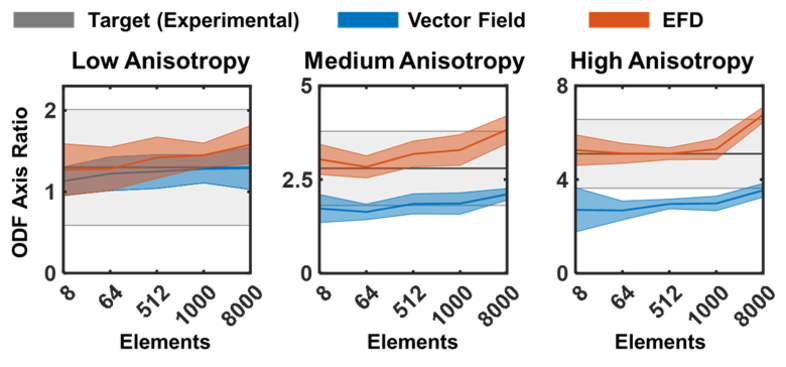

Supplement: S4 Fig — Directional guidance was generally unaffected by mesh refinement. A 1x1x1 mm3 cube was meshed between 8 and 8,000 FEBio hex8 elements. (TIF) [file pcbi.1011553.s006.tif]

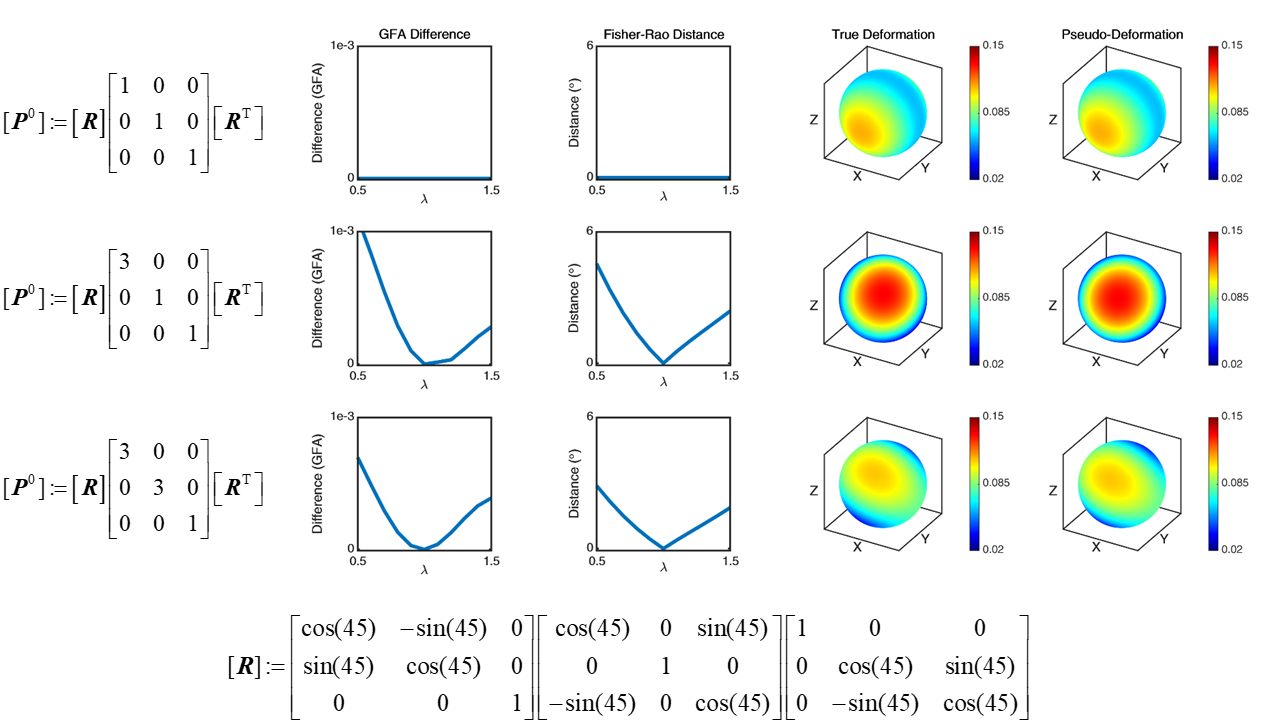

Supplement: S5 Fig — Three different initial EFDs were tested with the EFD designated by P0 (Supporting Methods E in S1 Text). P0 is an EFD representing a uniform, uniaxially aligned, or planarly isotropic (transversely anisotropic) EFD. These EFDs are rotated by R, a rotation matrix of 45° about the X, Y, and Z-axes. The rotation ensures that deformations do not occur along the EFD principal directions (in which cases the pseudo-deformation and “true deformation” are identical during tension and some shear cases). The resulting GFA for each are compared (deformation solid, pseudo-deformation dashed). The Fisher-Rao distance between ODFs was also calculated for each. The resulting ODFs are visualized via heat maps. Heat maps correspond to the highest level of stretch tested. (TIF) [file pcbi.1011553.s007.tif]

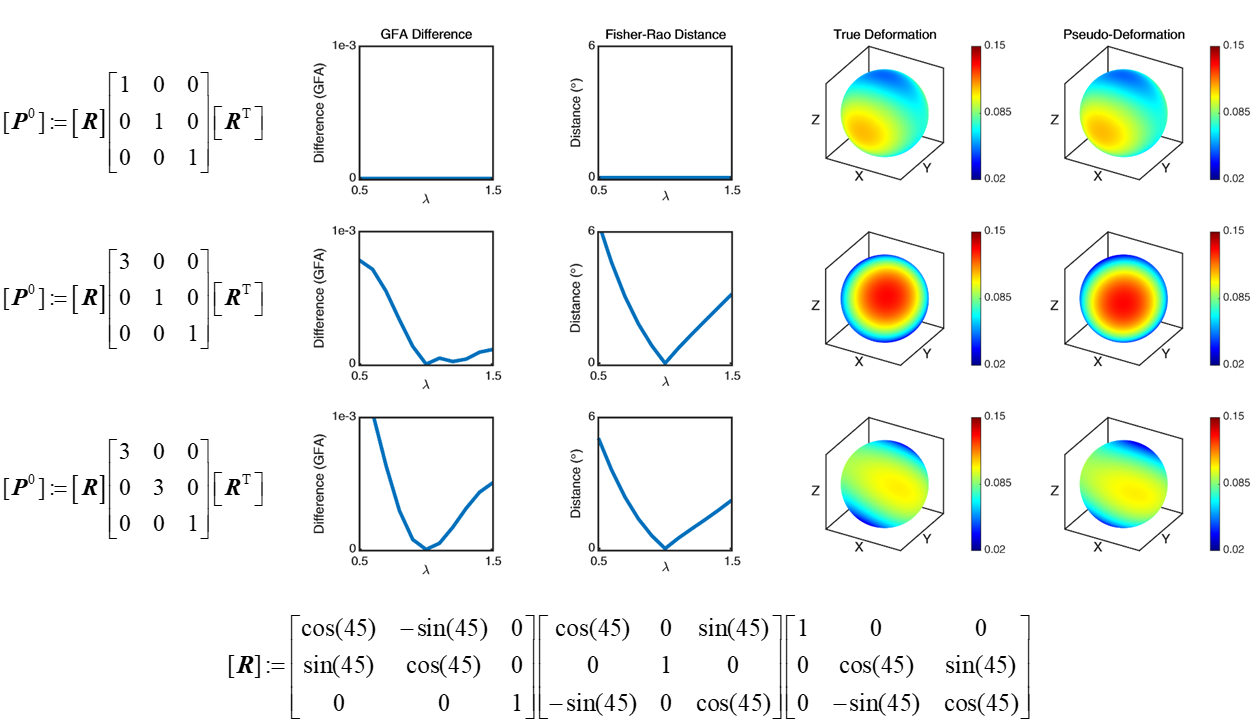

Supplement: S6 Fig — Three different initial EFDs were tested with the EFD designated by P0 (Supporting Methods E in S1 Text). P0 is an EFD representing a uniform, uniaxially aligned, or planarly isotropic (transversely anisotropic) EFD. These EFDs are rotated by R, a rotation matrix of 45° about the X, Y, and Z-axes. The rotation ensures that deformations do not occur along the EFD principal directions (in which cases the pseudo-deformation and “true deformation” are identical during tension and some shear cases). The resulting GFA for each are compared (deformation solid, pseudo-deformation dashed). The Fisher-Rao distance between ODFs was also calculated for each. The resulting ODFs are visualized via heat maps. Heat maps correspond to the highest level of stretch tested. (TIF) [file pcbi.1011553.s008.tif]

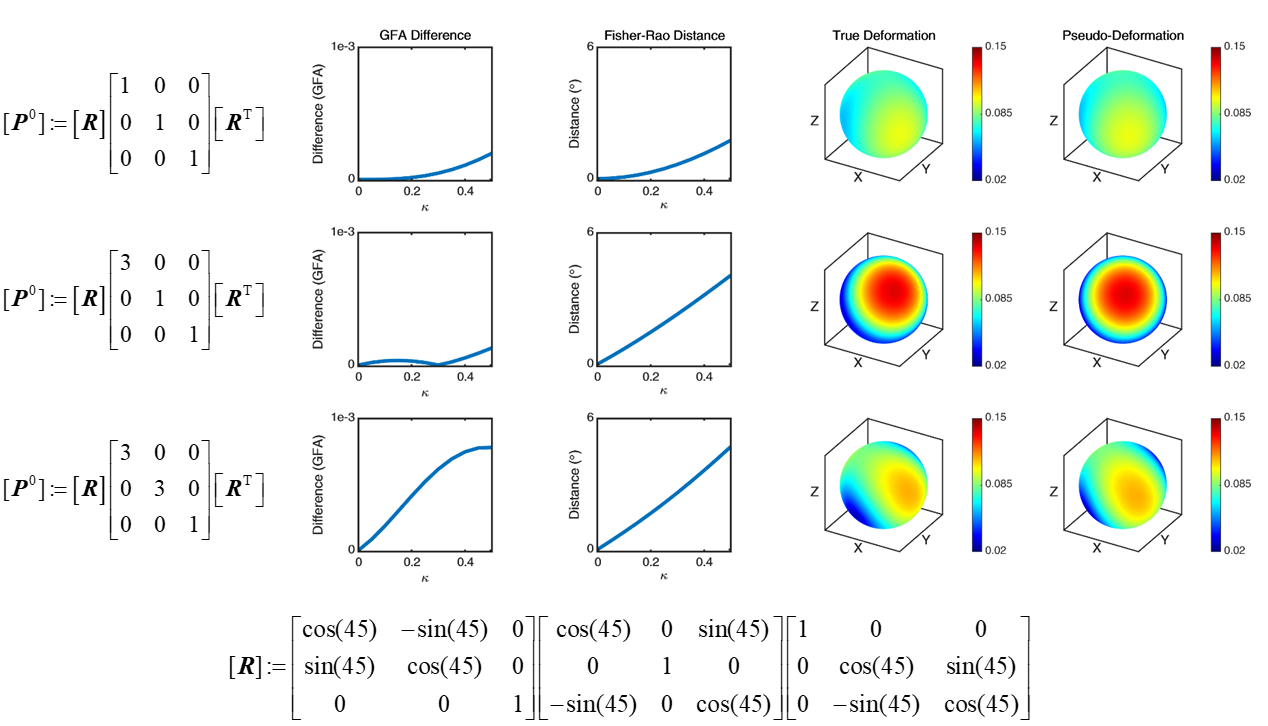

Supplement: S7 Fig — Three different initial EFDs were tested with the EFD designated by P0 (Supporting Methods E in S1 Text). P0 is an EFD representing a uniform, uniaxially aligned, or planarly isotropic (transversely anisotropic) EFD. These EFDs are rotated by R, a rotation matrix of 45° about the X, Y, and Z-axes. The rotation ensures that deformations do not occur along the EFD principal directions (in which cases the pseudo-deformation and “true deformation” are identical during tension and some shear cases). The resulting GFA for each are compared (deformation solid, pseudo-deformation dashed). The Fisher-Rao distance between ODFs was also calculated for each. The resulting ODFs are visualized via heat maps. Heat maps correspond to the highest level of stretch tested. (TIF) [file pcbi.1011553.s009.tif]

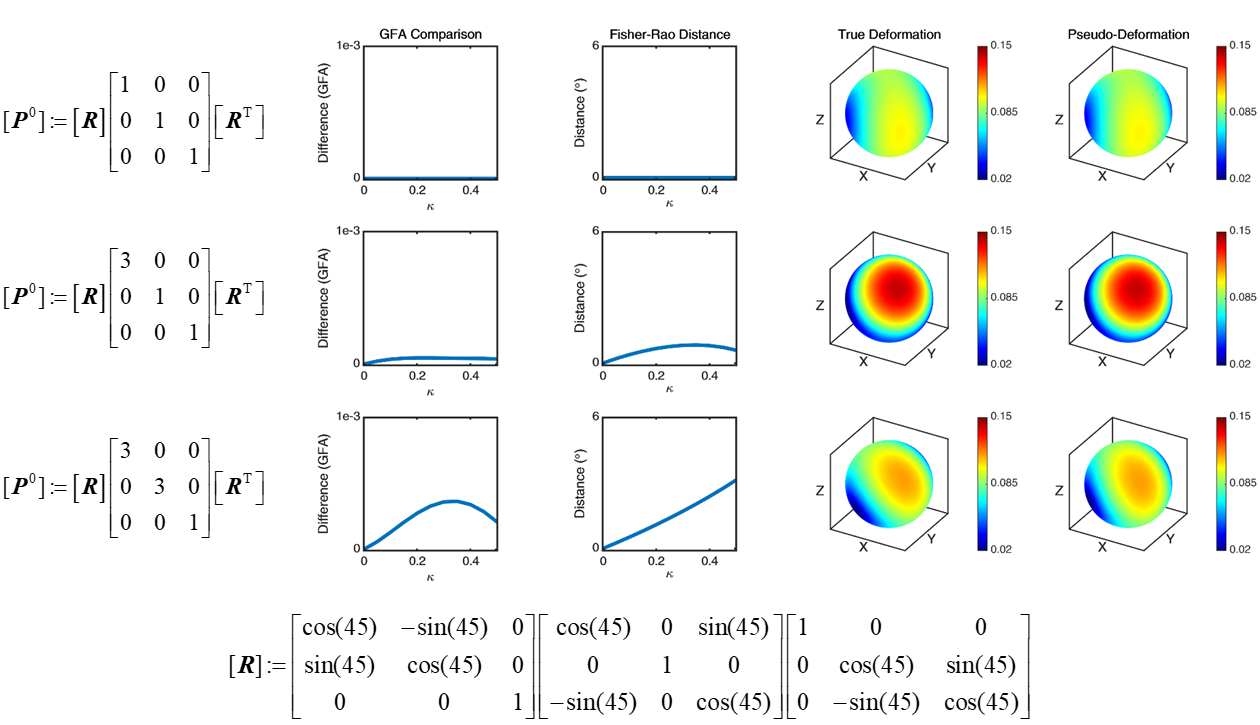

Supplement: S8 Fig — Three different initial EFDs were tested with the EFD designated by P0 (Supporting Methods E in S1 Text). P0 is an EFD representing a uniform, uniaxially aligned, or planarly isotropic (transversely anisotropic) EFD. These EFDs are rotated by R, a rotation matrix of 45° about the X, Y, and Z-axes. The rotation ensures that deformations do not occur along the EFD principal directions (in which cases the pseudo-deformation and “true deformation” are identical during tension and some shear cases). The resulting GFA for each are compared (deformation solid, pseudo-deformation dashed). The Fisher-Rao distance between ODFs was also calculated for each. The resulting ODFs are visualized via heat maps. Heat maps correspond to the highest level of stretch tested. (TIF) [file pcbi.1011553.s010.tif]

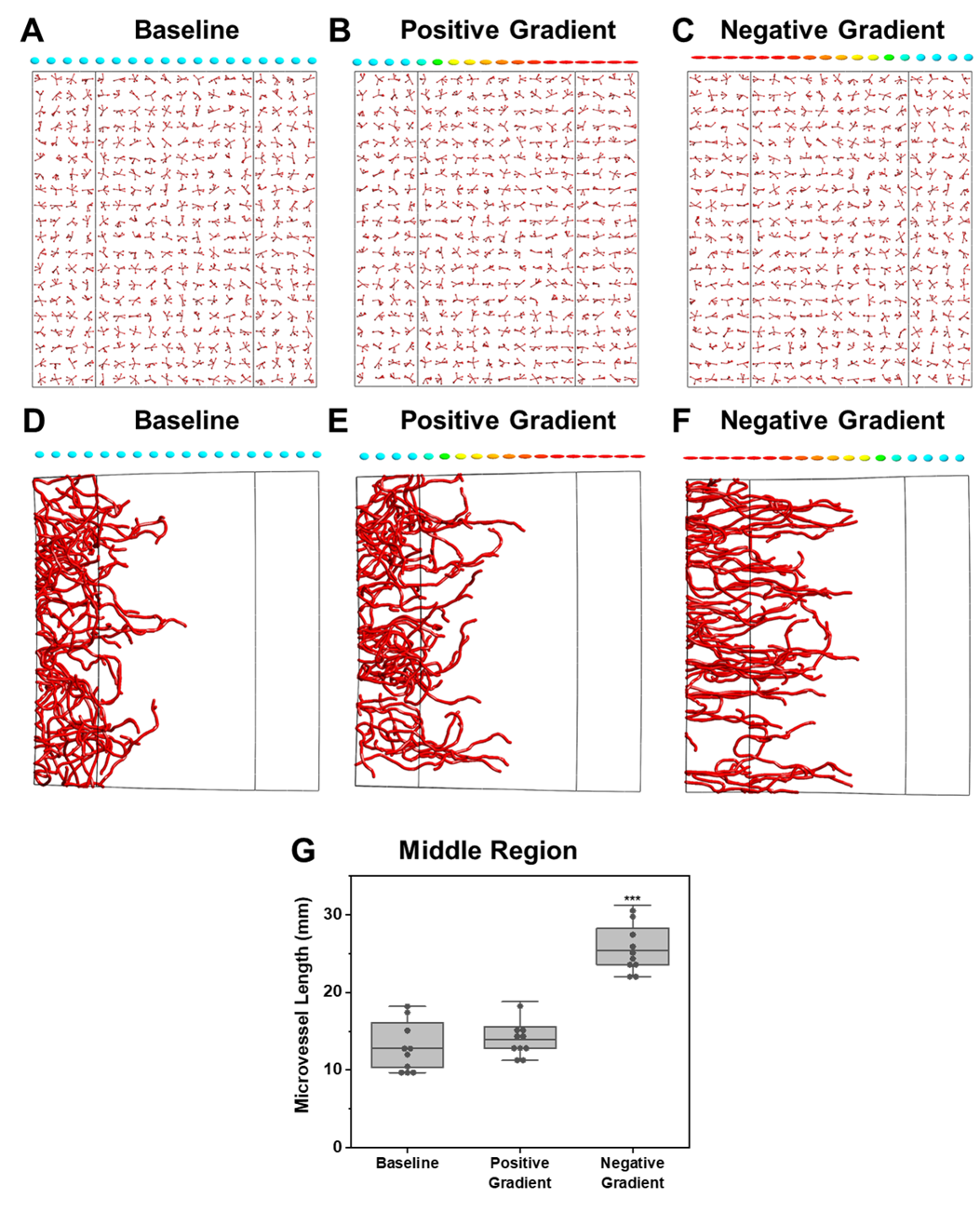

Supplement: S9 Fig — A-C. Top-down view of the initial fibril directions for each element. Directions were randomly sampled from the element’s collagen EFD. Local matrix alignment is indicated by ellipsoidal glyphs above each representative image with the color indicating the anisotropy (blue: low; red: high). D-F. Microvessels were seeded on the left end of a rectilinear domain. Growth was either simulated in a relatively isotropic matrix (baseline) or a matrix characterized by a positive or negative anisotropy gradient. Microvessels for all models failed to reach the far region on the right. EFD simulations, on the other hand, predicted vascularization of the far region for both gradient models. G. Differences were also observed for growth in the middle region. Here, only a negative gradient resulted in increased vascularization, differing from the EFD simulations where both gradients resulted in increased growth in the middle region. (*: p<0.001 w.r.t baseline; @: p<0.001 pairwise comparison, 1 way ANOVA with Sidakholm post hoc). (TIF) [file pcbi.1011553.s011.tif]

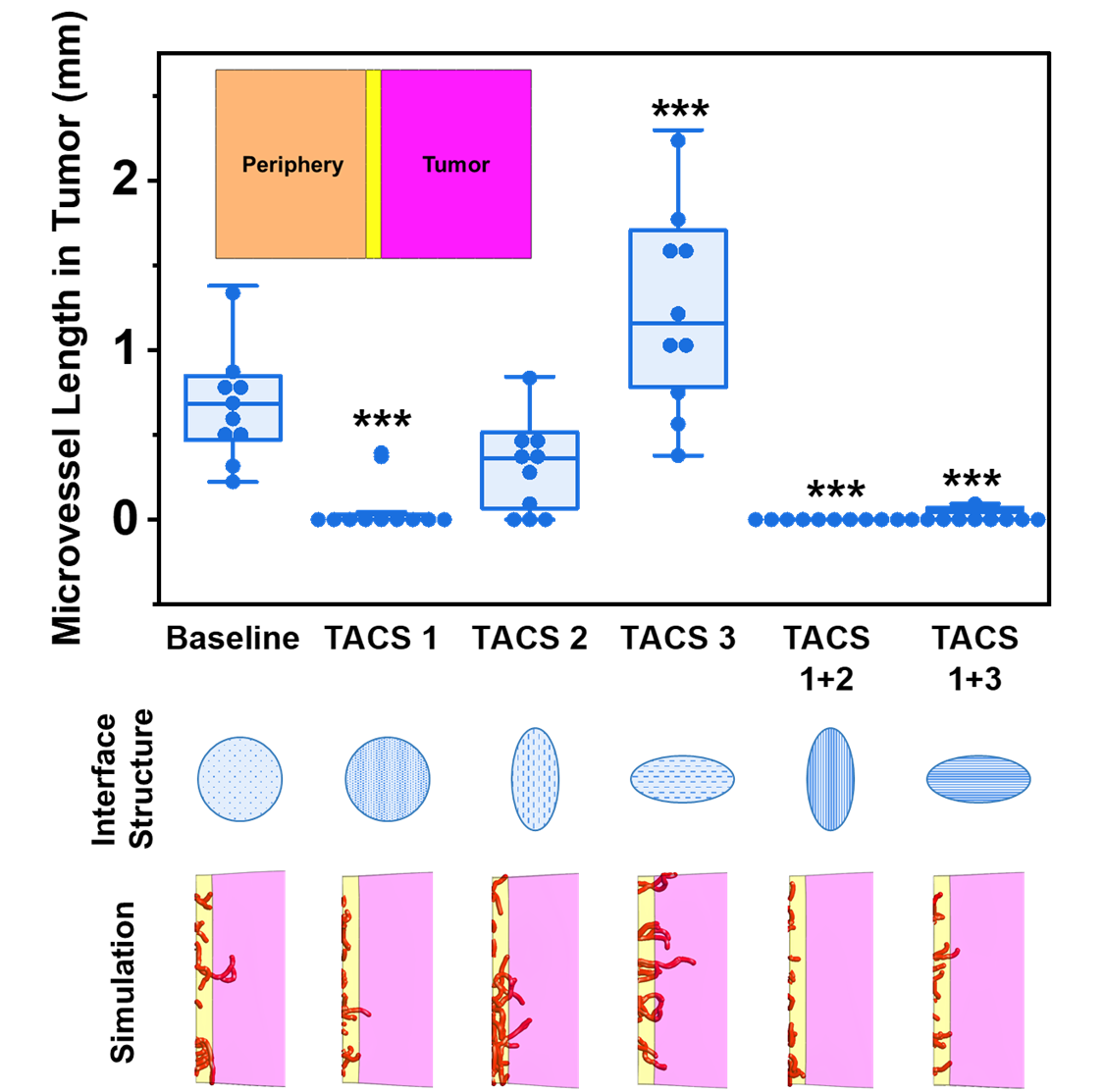

Supplement: S10 Fig — Microvessels were simulated to originate in a tumor periphery (inset, orange). Microvessels grow towards the tumor (inset, magenta) but must cross a tissue interface (inset, yellow). Alignment and density of the interface is varied to mimic various tumor associated collagen signatures (TACS). The interface structure contained either an isotropic fibril ODF (circle) or aligned ODFs (ellipses) running either perpendicular to the tumor or towards the tumor. The interface density was either 3 mg/mL or 5 mg/mL. Representative Z projections of the interface and tumor region are presented at the bottom. High interface density generally reduced the length of microvessels that crossed into the tumor. Fibril alignment towards the tumor facilitated crossing while alignment perpendicular to the tumor deflected vessels or trapped them within the interface. Unlike the EFD simulations, fibril alignment towards the tumor did not nullify the effects of increased matrix density for TACS-1+3. ***: p<0.001. 1 Way ANOVA with Sidakholm post hoc test. (TIF) [file pcbi.1011553.s012.tif]

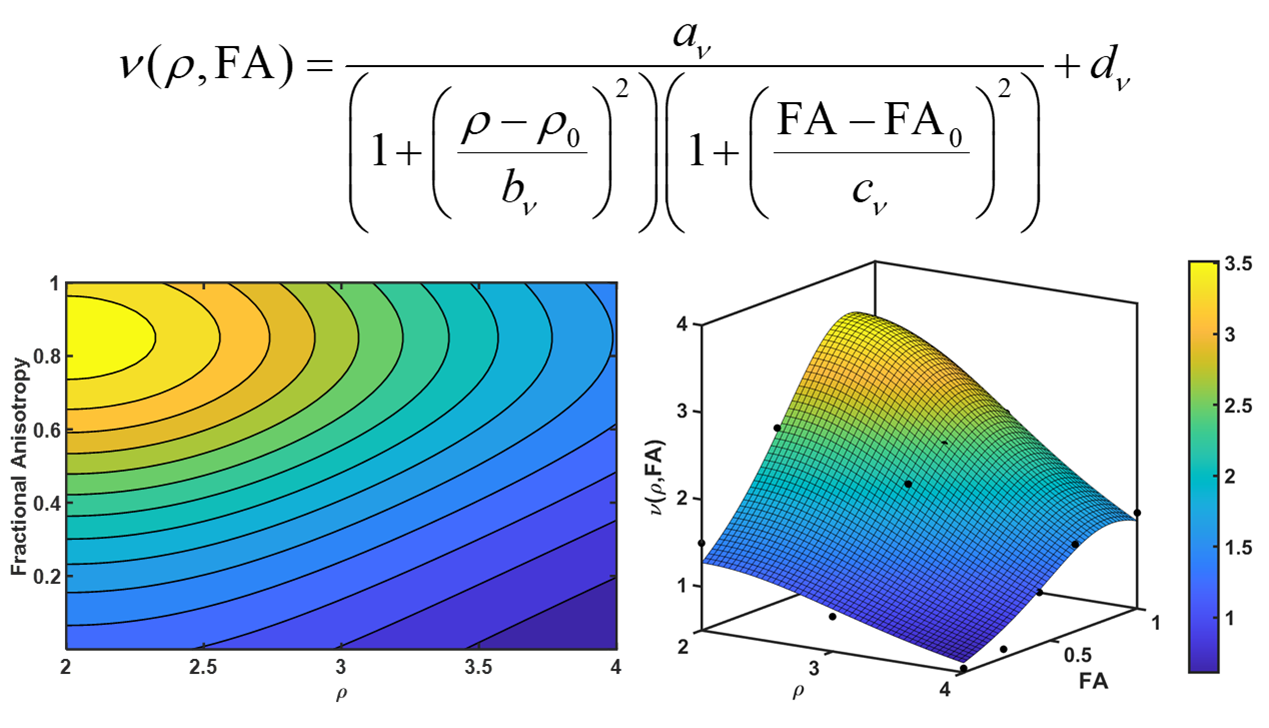

Supplement: S11 Fig — Eq [12] is a function designed to scale vessel growth based on matrix density and anisotropy. Scaling decreases velocity with increased matrix density (ρ) and increases with anisotropy (FA). Left: contour level plot with 0.2 level increments. Right: 3D contour plot with experimental points overlaid. (TIF) [file pcbi.1011553.s013.tif]

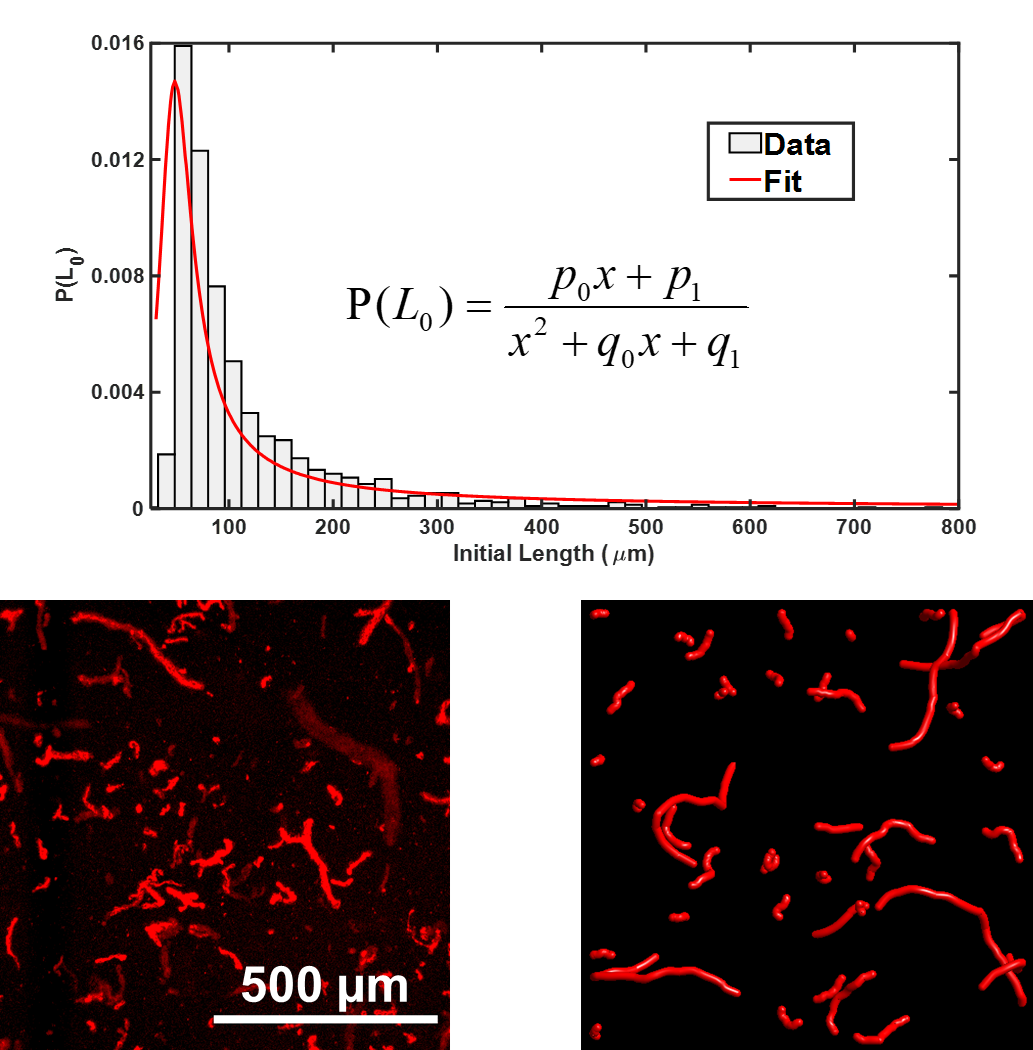

Supplement: S12 Fig — Top: The initial length of each discrete microvessel was sampled from a rational function P(L0) that had been fit to experimental measures of initial microvessel length. Bottom: Left: Representative confocal Z projection of microvessels at the beginning of culture. Right: Representative Z projection of initial microvessels in simulations. Depth of field for both images is 200 μm. (TIF) [file pcbi.1011553.s014.tif]

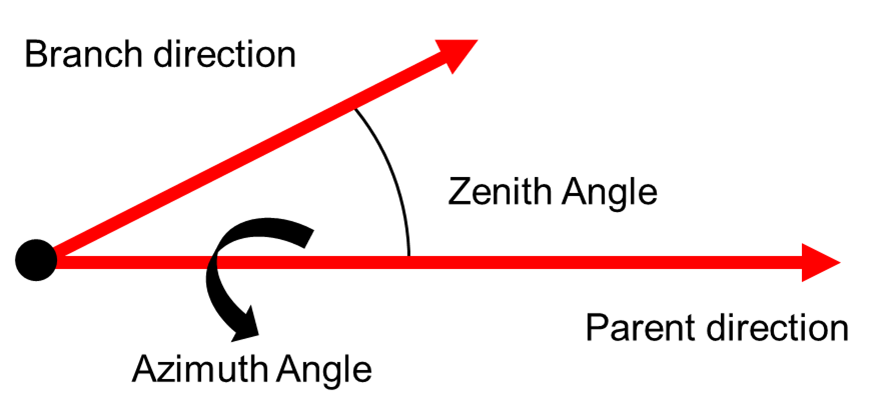

Supplement: S13 Fig — The direction of a new branch is determined by the direction of the parent microvessel, the zenith angle, and the azimuth angle. The zenith angle is the angle describing the elevation of the branch direction from the parent direction. The azimuth angle represents a rotation about the axis of the parent direction. (TIF) [file pcbi.1011553.s015.tif]

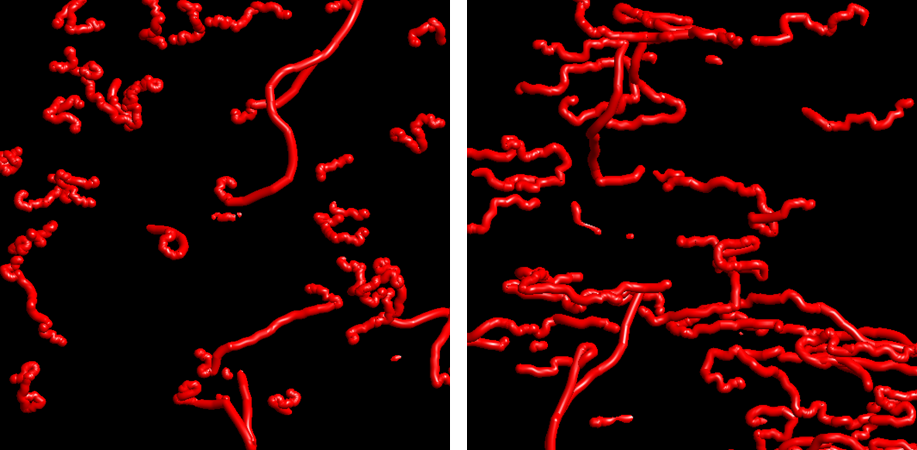

Supplement: S14 Fig — Z projections of microvascular networks grown in low (left) and high (right) anisotropy collagen using a continuous EFD method with the fibril weight α set to 0.9 (minimal persistence). Depth of field = 200 μm. High fibril weight leads to high curvature that resembles pathological angiogenesis. (TIF) [file pcbi.1011553.s016.tif]

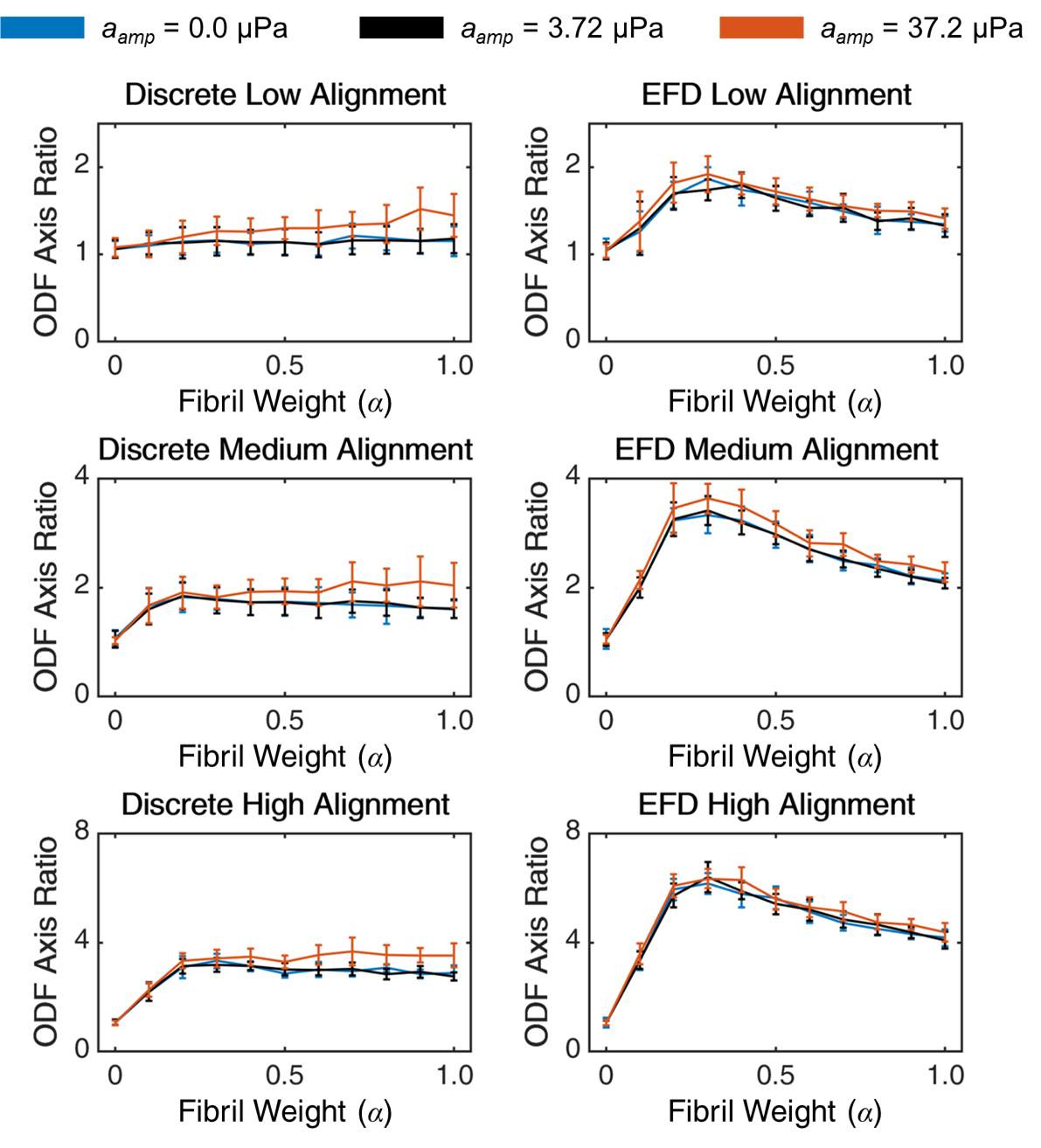

Supplement: S15 Fig — Low-density results: The sensitivity of the model predictions to the neovessel sprout stress magnitude (aamp) was studied across the full range of possible collagen orientation weights (α). The ratio of the 2 greatest ODF semiprincipal axes was plotted for each condition on the y-axis. The results for 3 mg/mL simulations are displayed with results from discrete fibril vector simulations on the left and results from EFD simulations on the right. No differences are observable between the stress-free (aamp = 0.0 μPa) and baseline stress (aamp = 3.72 μPa). In contrast, the high-stress case led to increased polarization of the vascular network for a number of cases, as indicated by increased values on the y-axis. These effects are visibly more pronounced for the discrete simulations. (TIF) [file pcbi.1011553.s017.tif]

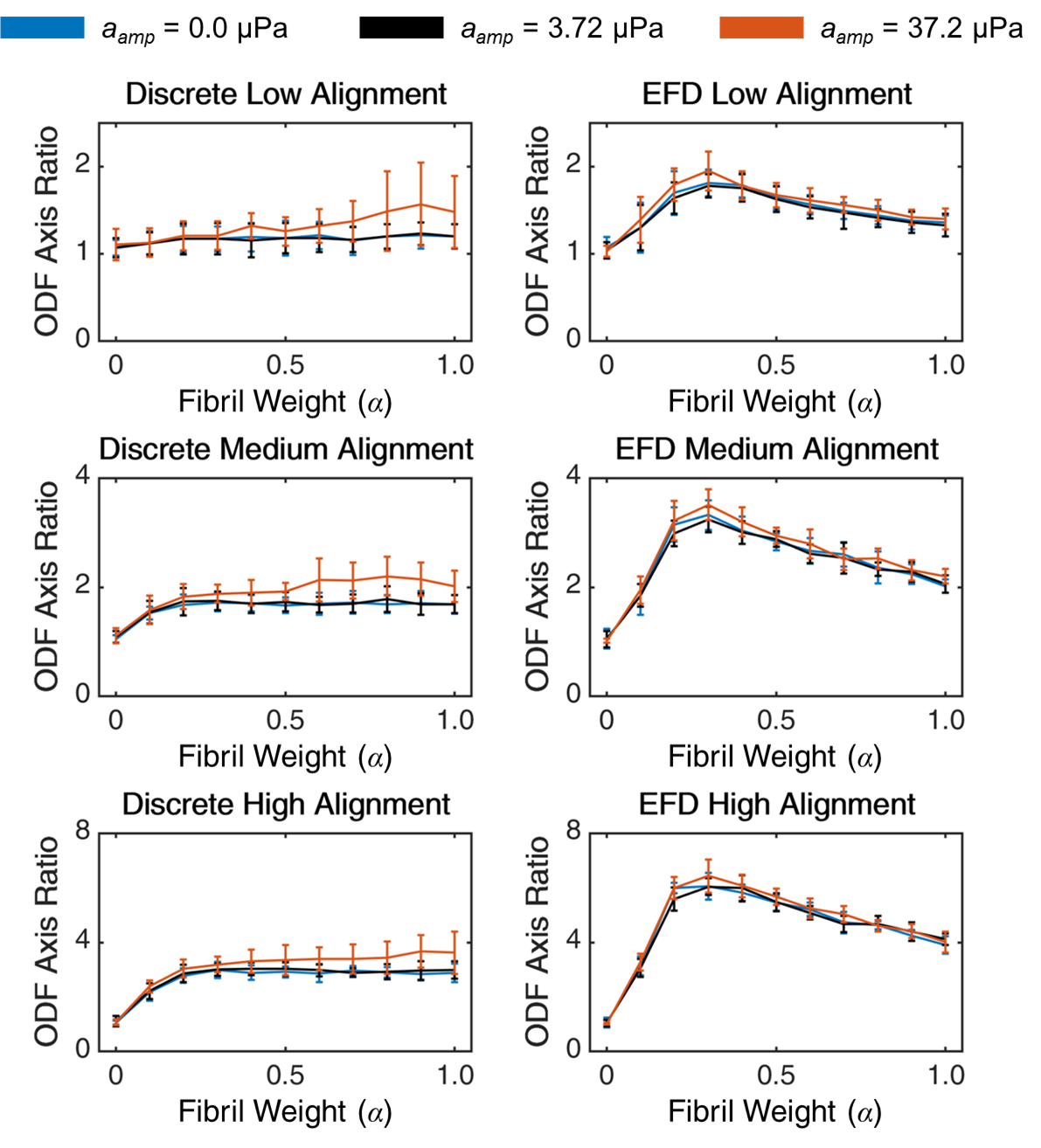

Supplement: S16 Fig — High-density results: The sensitivity of the model predictions to the neovessel sprout stress magnitude (aamp) was studied across the full range of possible collagen orientation weights (α). The ratio of the 2 greatest ODF semiprincipal axes was plotted for each condition on the y-axis. The results for 4 mg/mL simulations are displayed with results from discrete fibril vector simulations on the left and results from EFD simulations on the right. No differences are observable between the stress-free (aamp = 0.0 μPa) and baseline stress (aamp = 3.72 μPa). In contrast, the high-stress case led to increased polarization of the vascular network for a number of cases, as indicated by increased values on the y-axis. These effects are visibly more pronounced for the discrete simulations. (TIF) [file pcbi.1011553.s018.tif]
