# Supplementary material for: Spatial Configurations of 3D Extracellular Matrix Collagen Density and Anisotropy Simultaneously Guide Angiogenesis
Source: PLoS Comput Biol. 2023 Oct 23;19(10):e1011553. doi: 10.1371/journal.pcbi.1011553 (PMC10621972; doi:10.1371/journal.pcbi.1011553)
Supplement: Attachment — Submitted filename: comments.pdf [file pcbi.1011553.s019.pdf]

Referee report on "Spatial Configurations of 3D Extracellular Matrix Collagen Density and Anisotropy Simultaneously Guide Angiogenesis"

Remarks to the Authors:

This article presents a computational study of microvascular growth and directed movement influenced by ECM collagen anisotropy and density within the computational framework AngioFE. Ellipsoidal Fibril Distributions (EFDs) and vector fields model ECM collagen anisotropy. Qualitative agreement has been observed between simulations and former experimental results regarding microvascular network growth. Comparing produced Orientation Distribution Functions a good agreement is observed with experiments when EFDs are used which suggests that EFD is a promising of modeling ECM anisotropies. For convenience they connect deformations of EFDs with pseuso-deformation and they give some examples for the validity of their choice. In my opinion, it is a very interesting work and this computational tool could provide further exploration of neovascularization, potentially towards the avenue of suggesting new structures that facilitate or suppress microvascular growth by controlling the directed migration though mechanical cues.

Here one can notice that a lot of effort is needed to employ the developed models which increases the difficulty of readability due to the complex structure of the theory with a big number of adjustable parameters.

However, the authors should address the following points for the validity and readability of this work.

Major remarks:

- Text is not clear and confusing in some points. For instance, assumptions are not clear and scattered over the manuscript. Furthermore, some main assumptions are not referred at all in the text, but they are implied by the references. It will be valuable for potential users of AngioFE a clear presentation of the main assumptions and of the involved equations, both gathered in a section. This will increase readability and reproducibility. Furthermore, users will be able to control possible violations of the basic assumptions easier while a more self contained study will be presented. It will be helpful to refer the order these equations are solved, e.g which parts are solved as a system and which parts individually?
- **Line 154: EFDs rotated and scaled to approximate both symmetric and non-affine deformations that occur at the gel edges, center, and through the thickness.**

  How do you compute the scaling and rotation? Do you minimize some kind of distance between the rotated-scaled EFD and the deformation? Does pseudo-deformation get involved?

- **Line 156: To quantitatively verify our approach, we first calculated the difference in generalized fractional anisotropy (GFA) between deformed and pseudo-deformed distributions undergoing modes of tension, compression, and shear.**

  Here I am missing something, therefore i will try to describe what I have understood from the presented theory. In the given examples of uniaxial and biaxial tension-compression you provide two diagonal matrices, in supplementary equations (S7) and (S8), therefore F equals (S7) or (S8). In both cases F is diagonal. The reference ellipsoid Ω^o is given by the relation $x^o = P^o x^I$, where $x^I \in \Omega^I$ and Ω^I is a sphere, (line 369). Then, the ellipsoid Ω^o is mapped to the deformed configuration by the mapping $x : \Omega^o \to \Omega$ with deformation gradient $F = \partial x / \partial x^o$. You define the "true" deformation gradient as

  $$F^n = F P^o,$$

  but for the aforementioned examples F is diagoanl and for every comparison the provided P^o is diagonal as well, which means F^n is diagonal. Therefore, the rotation of the polar decomposition is the unit matrix and $F^p = F^n$, but you illustrate that $F^p \neq F^n$.

  Can you explain further how did you validate that F^p approximates F^n?

- **352: In order to maintain the symmetry properties of the EFDs after deformation, we assumed that the EFD remains ellipsoidal but allowed for affine deformation (finite rotation and stretching) of its principal radii of curvature.**

  This choice should be connected with some assumption. One could say that around a small enough neighborhood of a point, the deformation can be considered as homogeneous (affine) which means small enough EFD remains ellipsoidal. On the other hand under large deformations, deformation gradients can be discontinuous or with high variation at some areas of the matrix. In line 472 it is referred “The end of each line is a vessel tip that is capable of growing and contracting the surrounding matrix”. Here if the deformation under the microvascular growth remains small then the above assumptions sound reasonable.

- **359: Define SPD (symmetric positive definite).**

- **377: Replace $\hat{B}$ with B.**

- **419: The values of Ω were computed by calculating the percentage of points s that were contained within each triangular face on an undeformed icosahedron.**

  What do you mean by the values of Ω, do you mean deformed points? I don’t understand that point.

- **429:** For homogeneous deformations equation (8) is the same as equation (5). For non homogeneous deformations how do you compute (8)?

Minnor remarks:

- **Line 43: directional guidance (migration direction)**

  Is it better known as directed migration?

- **Line 83:** growth rates, proliferation (choose either to use growth rate or proliferation).

- **Line 119:** EFD is defined but used earlier in introduction (as EFDs) without clarification, see line 93. Definition must be moved there and thereafter used as EFDs.

- **Line 136: projected onto the XY and XZ planes (Fig. 2B).**

  Replace with projected onto the XY (Fig. 2B) and XZ planes (Figure 2B shows only XY plane).

- **Line 207: Growth was encouraged along directions ...**

  Maybe it's more appropriate as "Enhanced growth/migration was observed along directions"?

- Authors might consider the following papers which seem related with Tumor Associated Collagen Signatures (TASC), (Ray, A., & Provenzano, P. P. (2021), Current Opinion in Cell Biology, 72, 63-71) and (G. Grekas, et al. Journal of the Royal Society Interface 18.175 (2021): 20200823). Is it possible to incorporate studies similar to the latter in your computational framework?

- Authors should comment on the value of a model that was 30 or more adjustable parameters. More and more complex models emerge and my concern is related to reproducibility, robustness and readability regarding these theories.
