# Supplementary material for: Spatial Configurations of 3D Extracellular Matrix Collagen Density and Anisotropy Simultaneously Guide Angiogenesis
Source: PLoS Comput Biol. 2023 Oct 23;19(10):e1011553. doi: 10.1371/journal.pcbi.1011553 (PMC10621972; doi:10.1371/journal.pcbi.1011553)
Supplement: Attachment — Submitted filename: LaBelle_PLOS_CompBiol_ResponseToReviewerComments.pdf [file pcbi.1011553.s020.pdf]

We thank the reviewers for their time and insightful comments that have improved the clarity and quality of our manuscript. We have addressed all reviewer comments in point-by-point responses below. Of particular note, we added “Model Formulation” sections and a figure to clarify the simulation workflow. We also corrected the visualization of pseudo-deformation validation based on Reviewer #2’s comment, which pointed out data that did not match the equations in the text. We have also incorporated the reviewers’ feedback by including more clarifications on the methods and limitations of the study. Changes to the manuscript are marked with a line along the left margin and changed text is colored. Please note that we added new figure at the beginning (Fig 1, model formulation) so the figure numbers corresponding to reviewer comments should be incremented by 1.

Reviewer #1

This manuscript tackles a problem of current interest in mathematical modelling of angiogenesis, namely, how the growth of the vessel sprouts couples to the mechanics of the ECM. It is well known that such feedback (i.e. how the orientation of the fibers within the ECM guides the migration of ECs and the growth of the vessels, and how the vessels deform, realign and remodel the ECM) is crucial to understand the mechanisms of angiogenesis.

The authors present a methodology that allows them to quantify the anisotropy of the orientation distribution functions of the fibrils within the ECM. To do so they introduce the so-called ellipsoid fibril distribution (EDF) and an associated Monte-Carlo sampling method. The authors claim that this methodology allows them to improve the results obtained by means of previous approaches which introduced a vector field accounting for the average local orientation, since the latter does not contain enough information about the actual distribution of orientations.

We thank the reviewer for their kind comments. One clarification we emphasize is that cellular proliferation and microvascular extension rates increase with ECM collagen anisotropy. This is particularly relevant in pathologies that take advantage of modifications in ECM structure such as solid tumorigenesis.

While this is an interesting approach and the results look promising, the manuscript in its current form cannot be published and major revisions regarding the presentation of their model are required.

1. *The major concern involves the description of the model for angiogenesis. While the description of how rate the growth is influenced by both concentration of collagen and fibril orientation seems adequate, information about how the fibril orientation feeds into the model of angiogenesis is lacking. In line 477, the authors mention that "The direction a tip grows depends on a combination of persistence (prior tip direction) and fibril orientation". This needs to be further explained and substantiated, specifically regarding the EFD description and the Monte-Carlo sampling by giving the corresponding formulae (as it has been done for the rate of growth in Eq. 11). I suggest that the authors write a section on model formulation which clearly states which compartments are considered in the model, how each of this compartments*

is actually modelled, and, more importantly, how the different compartments are coupled and integrated within the whole model.

Thank you for the suggestion. We previously omitted these details in favor of citations with the intent of brevity. We agree that a "Model formulation" section will help to contextualize the advancements of this research. We have added both a brief overview to the Results (*AngioFE Model Formulation*, Page 8 Line118) as well as detailed description to the methods (*FEBio + AngioFE computational model of microvascular growth*, Page 28, Line 550) of the revised manuscript. We also added a figure detailing the simulation workflow (Fig 1 in revised manuscript).

2. *Without the information the accuracy/validity of claims such as "These differences arose due to the dependence of the growth rate on the local anisotropy; as neovessels grew up the positive gradient, the potency of matrix growth and guidance cues increased" (lines 263 to 265) cannot be assessed.*

   These observations are based on following the mathematical logic implied by the simulation rules. To further ensure these claims are accurate, we performed the predictive simulations (anisotropy gradients, TACS) with the prior vector field framework. The results of those simulations were added on Page 13, Lines 232-237 and Page 14, Lines 262-265 of the revised manuscript.

3. *Another issue that should be taken into account is that the concept of the "orientation distribution function" is very informally defined. While the level of information provided is probably fine for a journal with a more specialised readership, a more detailed introduction to this concept should make the paper more accessible to a more general readership.*

   Thank you, we have added additional background information to Page 6, Lines 81-84.

4. *The acronym "SPD" does not seem to be defined.*

   This acronym is now defined at first usage on Page 20 Line 382.

5. *Lines 214 to 216: "As a result, these simulations were able to accurately predict anisotropic guidance in agreement with prior experimental measures. In contrast, simulations using the vector field approach under-predicted anisotropic guidance." What is the significance of this "under-prediction"? For example, could the simpler model reproduce some of the features in Figs. 4b and 4c?*Author note: 5b and 5c in the revised manuscript.*

   In our calibration models, the vessels from vector field simulations did not reorient along the X axis to a similar degree as vessels from EFD fields (Figs. 3, S3). To further demonstrate these limitations, we added vector field simulations of the predictive simulations (Results, Fig. S9-S10). Two important distinctions emerge. Please see Page 15, Lines 273-275.

Reviewer 2

This article presents a computational study of microvascular growth and directed movement influenced by ECM collagen anisotropy and density within the computational framework AngioFE. Ellipsoidal Fibril Distributions (EFDs) and vector fields model ECM collagen anisotropy. Qualitative agreement has been observed between simulations and former experimental results regarding microvascular network growth. Comparing produced Orientation Distribution Functions a good agreement is observed with experiments when EFDs are used which suggests that EFD is a promising of modeling ECM anisotropies. For convenience they connect deformations of EFDs with pseusodeformation and they give some examples for the validity of their choice. In my opinion, it is a very interesting work and this computational tool could provide further exploration of neovascularization, potentially towards the avenue of suggesting new structures that facilitate or suppress microvascular growth by controlling the directed migration though mechanical cues. Here one can notice that a lot of effort is needed to employ the developed models which increases the difficulty of readability due to the complex structure of the theory with a big number of adjustable parameters. However, the authors should address the following points for the validity and readability of this work.

We appreciate the reviewer's compliments but also acknowledge that the specialized methods to simulate taxis and directed cellular migration in response to matrix structure may overshadow the biological repercussions of the research. We have taken this in mind while addressing their specific comments.

1. *Text is not clear and confusing in some points. For instance, assumptions are not clear and scattered over the manuscript. Furthermore, some main assumptions are not referred at all in the text, but they are implied by the references. It will be valuable for potential users of AngioFE a clear presentation of the main assumptions and of the involved equations, both gathered in a section. This will increase readability and reproducibility. Furthermore, users will be able to control possible violations of the basic assumptions easier while a more self-contained study will be presented. It will be helpful to refer the order these equations are solved, e.g which parts are solved as a system and which parts individually?*

Thank you for these suggestions, Reviewer 1 had a similar suggestion. All assumptions and relevant equations are now organized in Model Formulation sections. We have added both a brief overview to the Results (*AngioFE Model Formulation*, Page 8 Line118) as well as detailed description to the methods (*FEBio + AngioFE computational model of microvascular growth*, Page 28, Line 550) of the revised manuscript. We also added a figure detailing the simulation workflow (Fig 1 in revised manuscript).

2. *Line 154: EFDs rotated and scaled to approximate both symmetric and non-affine deformations that occur at the gel edges, center, and through the thickness. How do you compute the scaling and rotation? Do you minimize some kind of distance between the rotated-scaled EFD and the deformation? Does pseudo-deformation get involved?*

Yes, the EFDs are "pseudo-deformed". Scaling and rotation is derived from the pseudo-deformed EFD by performing the eigendecomposition (spectral decomposition, equation (1)) of the pseudo-deformed tensor representing the EFD.

3. *Line 156: "To quantitatively verify our approach, we first calculated the difference in generalized fractional anisotropy (GFA) between deformed and pseudo-deformed distributions undergoing modes of tension, compression, and shear." Here I am missing something, therefore I will try to describe what I have understood from the presented theory. In the given examples of uniaxial and biaxial tension-compression you provide two diagonal matrices, in supplementary equations (S7) and (S8), therefore F equals (S7) or (S8). In both cases* $\boldsymbol{F}$ *is diagonal. The reference ellipsoid* Ω^0 *is given by the relation* $\boldsymbol{x}^0 = \boldsymbol{P}^0\boldsymbol{x}^I$, *where* $\boldsymbol{x}^I \in \Omega^I$ *and* Ω^I *is a sphere, (line 369). Then, the ellipsoid* Ω^0 *is mapped to the deformed configuration by the mapping* $\boldsymbol{x}: \Omega^0 \rightarrow \Omega$ *with deformation gradient* $\boldsymbol{F} = \partial\boldsymbol{x}/\partial\boldsymbol{x}^0$. *You define the "true" deformation gradient as* $\boldsymbol{F}^n = \boldsymbol{F}\boldsymbol{P}^0$, *but for the aforementioned examples* $\boldsymbol{F}$ *is diagonal and for every comparison the provided* $\boldsymbol{P}^0$ *is diagonal as well, which means* $\boldsymbol{F}^n$ *is diagonal. Therefore, the rotation of the polar decomposition is the unit matrix and* $\boldsymbol{F}^p = \boldsymbol{F}^n$, *but you illustrate that* $\boldsymbol{F}^p \neq \boldsymbol{F}^n$. *Can you explain further how did you validate that* $\boldsymbol{F}^p$ *approximates* $\boldsymbol{F}^n$*?*

The reviewer is correct here; we appreciate the reviewer's attention to detail. We agree that $\boldsymbol{F}^p = \boldsymbol{F}^n$ for cases where deformation occurs along the semiprincipal axes of the ellipsoid or the ellipsoid is isotropic. There was a mistake in our code that generated minor differences between the "true" and pseudo-deformed deformations for these cases. We previously recognized this and thus rotated the initial ODF $\boldsymbol{P}^0$ prior to deformation to evaluate the validity of pseudo-deformation when shearing and rotational strains occur. We have updated the figures (Figure 4 and Supplemental Figures 5-8) and text related to this content which should be mathematically consisted with the figures in the supporting text. The MATLAB code on our github repository is also updated and reproduces the revised figures.

4. *Line 352: "In order to maintain the symmetry properties of the EFDs after deformation, we assumed that the EFD remains ellipsoidal but allowed for affine deformation (finite rotation and stretching) of its principal radii of curvature." This choice should be connected with some assumption. One could say that around a small enough neighborhood of a point, the deformation can be considered as homogeneous (affine) which means small enough EFD remains ellipsoidal. On the other hand under large deformations, deformation gradients can be discontinuous or with high variation at some areas of the matrix. In line 472 it is referred "The end of each line is a vessel tip that is capable of growing and contracting the surrounding matrix". Here if the deformation under the microvascular growth remains small then the above assumptions sound reasonable.*

Thank you, we have clarified this assumption on Page 22, Lines 424-429, as well as Page 23, Line 439 of the revised manuscript.

5. *Line 359: Define SPD (symmetric positive definite).*

This acronym is now defined at first usage on Page 20 Line 382.

6. *Line 377: Replace* $\hat{B}$ *with B.*

$\boldsymbol{B}$ corresponds to the left Cauchy deformation tensor, which is calculated from active deformation. $\hat{\boldsymbol{B}}$ is an analog of $\boldsymbol{B}$. The distinction is that $\boldsymbol{B}$ reflects the deformation between the initial and final states ($\boldsymbol{x}^0$ to $\boldsymbol{x}$) while $\hat{\boldsymbol{B}}$ reflects the transformation from a unit sphere to the final state ($\boldsymbol{x}^i$ to $\boldsymbol{x}$). To avoid confusion with the standard left Cauchy deformation tensor, we leave the notation as $\hat{\boldsymbol{B}}$ on line 377. The $\boldsymbol{B}$ in equation (3) on line 379 has been changed to $\hat{\boldsymbol{B}}$. The text has been clarified on Page 24, Lines 460-462.

7. *Line 419: The values of Ω were computed by calculating the percentage of points* **s** *that were contained within each triangular face on an undeformed icosahedron. What do you mean by the values of Ω, do you mean deformed points? I don't understand that point.*

Thank you for this comment. Here, we are calculating the values of the 3D probability density function mapped onto an icosahedron. This has been clarified in the text, please see Page 26, Lines 501-502 of the revised manuscript.

8. *Line 429: For homogeneous deformations equation (8) is the same as equation (5). For non-homogeneous deformations how do you compute (8)?*

Equation (5) calculates the GFA from β_i, the eigenvalues (semiprincipal axes) of a 3D distribution undergoing homogenous deformations. Equation (8) is the generalized form of equation (5). Here, the standard deviation and rms are calculated for the values of the probability distribution function of the ODF Ω. The notation has been clarified, please see Page 26, Line 514 of the revised manuscript.

9. *Line 43: directional guidance (migration direction). Is it better known as directed migration?*

Here we consider "directional guidance" and taxis to be synonymous. This has been clarified, please see Page 5, Line 43 of the revised manuscript.

10. *Line 83: growth rates, proliferation (choose either to use growth rate or proliferation).*

Our intention was to distinguish extension rate (new length of vessel) and proliferation (cellular reproduction). "Growth rates" has been changed to "neovessel extension rate". Please see Pages 6-7, Lines 86-87 of the revised manuscript.

11. *Line 119: EFD is defined but used earlier in introduction (as EFDs) without clarification, see line 93. Definition must be moved there and thereafter used as EFDs.*

This acronym definition has been moved to the first usage in the Introduction. Please see Page 7, Line 99 of the revised manuscript.

12. *Line 136: projected onto the XY and XZ planes (Fig. 2B). Replace with projected onto the XY (Fig. 2B) and XZ planes (Figure 2B shows only XY plane).*

The figure callout was moved to indicate that only the XY data are visualized in the manuscript. Please see Page 10, Line 175.

13. *Line 207: Growth was encouraged along directions ... Maybe it's more appropriate as "Enhanced growth/migration was observed along directions"?*

We agree with the suggested change in tone and have made this revision. Please see Page 16, Lines 299-300 of the revised manuscript.

14. *Authors might consider the following papers which seem related with Tumor Associated Collagen Signatures (TASC), (Ray, A., & Provenzano, P. P. (2021), Current Opinion in Cell Biology, 72, 63-71) and (G. Grekas, et al. Journal of the Royal Society Interface 18.175 (2021): 20200823). Is it possible to incorporate studies similar to the latter in your computational framework?*

Thank you for providing the citations. The content of the first paper is generally covered in our other citations of those authors, but we have added the suggested citation, please see Page 13, Lines 241-242 of the revised manuscript.

The second paper suggested by the reviewer is very interesting but outside the scope of the current manuscript. We have plans to generalize this computational framework to simulate other migratory cell types. The study presented by the reviewer would be an appealing example.

15. *Authors should comment on the value of a model that was 30 or more adjustable parameters. More and more complex models emerge and my concern is related to reproducibility, robustness and readability regarding these theories.*

Please understand that the 30+ parameters listed in the supplement are not all used in each simulation. Many of the parameters in the table are restated for each unique modeling condition (e.g., the EFD parameters are unique for different individual models and a single EFD is described by 3 parameters.) Most articles published in PLOS Comp Biol contain far more than 30 parameters (some on the order of 100+).

The mechanical model itself consists of 9 parameters, which is very compact for representing biphasic, nonlinear, anisotropic, viscoelastic solids. The mechanical model parameters used in our models were derived from standard mechanical experiments that have been thoroughly validated and published (computational models of many biomechanical tests are provided on FEBio's repository).

Reviewer 3

Reviewer #3: I am sorry for this very short review due to time constraints. This an elegant approach to model the effect of ECM anisotropy on angiogenesis.

1. *I am sorry if I missed it, but it seems that in this version there is only a one-way feedback of the matrix orientations on endothelial cell migration. Then external strains can lead to ECM remodeling. However, traction on the matrix by migrating endothelial cells would also*

remodel the matrix. Is this part of the model already, or could the authors comment on how this can be incorporated in the model in the future?

Yes, this is already included in the modeling framework. The presented models are two-way feedback where external strains and cellular traction both lead to ECM reorientation and remodeling. This was mentioned briefly on lines 476-477 of the original manuscript. We have included additional information on this topic and we now explicitly described the two-way feedback relationship. Please see the newly added model formulation sections.

2. *At present it seems there is only qualitative comparison between models and experiment. Could a more quantitative comparison be provided? Also how does matrix orientation affect cell migration velocity in vitro and in silico?*

Quantitative comparisons are made in a several places in the manuscript. The calibration simulations were performed to optimize parameters so that simulated neovessel tip extension rate matched prior experiments (Page 31, Lines 603-605, Supplement) and neovessel tip reorientation matched published experiments (Fig 2, 3B, Supplement).

We now mentioned cell deflection in vitro due to structural barriers between adjacent tissues and associated citations, please see Page 19, Lines 361-366 of the revised manuscript.

The effects of matrix orientation on cell migration velocity are detailed in the Introduction and Discussion. Please see Page 5, Lines 58-62, as well as Page 18 Lines 347-349 of the revised manuscript.
