# Supplementary material for: Spatial Configurations of 3D Extracellular Matrix Collagen Density and Anisotropy Simultaneously Guide Angiogenesis
Source: PLoS Comput Biol. 2023 Oct 23;19(10):e1011553. doi: 10.1371/journal.pcbi.1011553 (PMC10621972; doi:10.1371/journal.pcbi.1011553)
Supplement: Attachment — Submitted filename: comments.pdf [file pcbi.1011553.s021.pdf]

Referee report on "Spatial Configurations of 3D Extracellular Matrix Collagen Density and Anisotropy Simultaneously Guide Angiogenesis" (PCOMPBIOL-D-23-00055R1)

Remarks to the Authors:

In the revised manuscript the main issues about readability and reproducibility have been improved. Even if I would prefer more formulas and a better description of the assumptions I believe the revised version is appropriate for publication in PLOS Comp. Biol. under few clarifications-corrections as they described below.

Remarks:

- A definition, a formula or some kind of reference regarding ODF would be useful.
- **Line 24:** "generalized to investigate" instead of "generalized investigate".
- **Line 171:** "corresponded" to "correspond".
- **Line 365:** Do you mean TACS-2 than TACS-1+2 instead of TACS-2 and TACS-1+2?
- **Line 460:** B given from equation (3) should be replaced by $\hat{B}$. In line 463 it is mentioned that B transforms points from x^0 to x and $\hat{B}$ points x^i to x. From picture 7 one deduces that $B = FF^T$ and $\hat{B} = F^n(F^n)^T$.
