# Supplementary material for: Spatial Configurations of 3D Extracellular Matrix Collagen Density and Anisotropy Simultaneously Guide Angiogenesis
Source: PLoS Comput Biol. 2023 Oct 23;19(10):e1011553. doi: 10.1371/journal.pcbi.1011553 (PMC10621972; doi:10.1371/journal.pcbi.1011553)
Supplement: Attachment — Submitted filename: LaBelle_PLOS_Comp_Biol_R2_ResponseToReviewerComments.pdf [file pcbi.1011553.s022.pdf]

We again thank the reviewers for their time and comments. We have addressed all reviewer comments in point-by-point responses below. We have added additional supplementary information describing the sensitivity of the model to the magnitude of the traction as suggested by Reviewer 3. Changes to the manuscript are marked with a line along the left margin and changed text is colored.

Reviewer #1

The authors have made a thorough revision of the paper in response to the reviewers comments.

We thank reviewer 1 and appreciate their prior comments that helped us clarify the manuscript.

Reviewer 2

In the revised manuscript the main issues about readability and reproducibility have been improved. Even if I would prefer more formulas and a better description of the assumptions I believe the revised version is appropriate for publication in PLOS Comp. Biol. under few clarifications-corrections as they described below.

We appreciate reviewer 2's additional comments and consistent attention to detail during the review process. We address the specific comments below:

1. *A definition, a formula or some kind of reference regarding ODF would be useful.*

   We have added a mathematical definition of the ODF and expanded contextual information in the manuscript. Please see lines 84-100 of the revised manuscript.

2. *Line 24: "generalized to investigate" instead of "generalized investigate".*

This change has been made with slight modification in-text ("generalized to investigate other mechanobiological relationships" changed to "generalized to other mechanobiological relationships").

3. *Line 171: "corresponded" to "correspond".*

Change made in place.

4. *Line 460:* $\boldsymbol{B}$ *given from equation (3) should be replaced by* $\boldsymbol{\tilde{B}}$*. In line 463 it is mentioned that* $\boldsymbol{B}$ *transforms points from* $\boldsymbol{x}^0$ *to* $\boldsymbol{x}$ *and* $\boldsymbol{\tilde{B}}$ *points* $\boldsymbol{x}_i$ *to* $\boldsymbol{x}$*. From picture 7 one deduces that* $\boldsymbol{B} = \boldsymbol{F}\boldsymbol{F}^T$ *and* $\boldsymbol{\tilde{B}} = \boldsymbol{F}_n(\boldsymbol{F}_n)^T$ *.*

The reviewer is correct. The equation was not correctly converted when our pdf was generated. We have ensured that the equations are set appropriately in the resubmitted pdf. We again thank the reviewer for their attention to detail.

Reviewer 3

1. *Regarding my comment #1 on the feedback between vessel tip traction and ECM reorientation, we get little insight as to what extent this aspect of the model affects the results. To what extent does it matter (in the model and also in the real system) that there is a cross-talk between vessel tip traction and ECM reorientation? In its present form the study seems to focus entirely on the effect of ECM structure on cell migration/angiogenesis. This is fine, but this limitation of the*

study should be clarified.

I would therefore recommend that the authors either:

(a) Include a new figure comparing the behavior of the model with and without vessel tip traction to give more insight into the extent that this important feature affects the model predictions; or

(b) Clarify the limitation of the study: i.e. discuss how it currently studies the effect of ECM structure on angiogenesis, and how it could be extended to get more insights in the two-way cross-talk between endothelial cells (ECs) and ECMs, and/or discuss if and why the effect of ECM structure on EC migration (one-way) is the most important.

We appreciate reviewer 3's curiosity in this area. We have previously established this relationship in a number of publications. Specifically, these prior publications compared changes in matrix orientation due to vascular growth in geometrically confined collagen gels. Please see:

- Underwood et al 2014, *Am. J. Physiol. Heart Circ. 2014*. Doi: 10.1152/ajpheart.00995.2013
- Edgar, Hoying, & Weiss 2015, *Ann. Biomed. Eng.* Doi: 10.1007/s10439-015-1334-3
- Edgar et al. 2015, *Biomech. Model. Mechanobiol.* Doi: 10.1007/s10237-014-0635-z

In these publications, we were able to approximate the neovessel traction stress (a_{amp} in the manuscript under consideration) by varying a_{amp} until FE simulations of neovessel-induced contractions in collagen gels matched experimental results. We found that setting $a_{amp} \approx 3.72$ μPa sufficiently predicted tissue-level deformations.

It is difficult to compare our prior data to direct measures of cellular traction in the literature (e.g., traction force microscopy, micropillar arrays). Nevertheless, we are confident this value is appropriate since reported values from cell experiments are derived from fundamentally different experimental models (e.g., forces due to individual attachments of micropillar assays, localized force calculation during traction force microscopy, etc). Further, we model traction as a polarized stress field – thus, the local forces experienced by the matrix vary by distance and orientation from the vessel tip. Please see Boys & Owens 2021, *APL Materials* for additional limitations in comparison of experimentally measured values (doi: https://doi.org/10.1063/5.0040953).

To address reviewer 3's comment, we performed a sensitivity study to determine how sprout tractions influenced the evolution of the vascular network for both discrete and EFD approaches. Specifically, we repeated the parametric study from the section titled *Comparison of discrete and continuous fibril distribution approaches*, with 3 different values of sprout traction magnitude. Discrete and EFD approaches were studied with sprout traction values of $a_{amp} = 0$ μPa, 3.72 μPa (baseline value used in prior publications), and 37.2 μPa (1 order of magnitude greater than the baseline value, likely supraphysiological). The results indicate that the resulting vascular network is insensitive to low levels of stress ($a_{amp} \leq 3.72$ μPa). In contrast, the vascular network hyperpolarizes in a positive feedback cycle due to higher values of traction stress. Similar trends may occur if the number of vessels was increased rather than the traction magnitude. We report these finding in the revised manuscript at lines 202-206, lines 314-320, and in Supporting Methods 13 and Figs. S15-S16.

2. *The authors have answered comment #2 on the quantitative comparison between model and experiment satisfactorily.*

Thank you.
